# Diatomite-Metal-Organic Framework Composite with Hierarchical Pore Structures for Adsorption/Desorption of Hydrogen, Carbon Dioxide and Water Vapor

**DOI:** 10.3390/ma13214700

**Published:** 2020-10-22

**Authors:** Gaofeng Wang, Elizabeth Graham, Shuilin Zheng, Jianxi Zhu, Runliang Zhu, Hongping He, Zhiming Sun, Ian D. R. Mackinnon, Yunfei Xi

**Affiliations:** 1 Institute for Future Environments and Science and Engineering Faculty, Queensland University of Technology (QUT), Brisbane, Queensland 4001, Australia; wanggaofeng@gig.ac.cn (G.W.); ian.mackinnon@qut.edu.au (I.D.R.M.); 2 CAS Key Laboratory of Mineralogy and Metallogeny, Guangdong Provincial Key Laboratory of Mineral Physics and Material Research and Development, Guangzhou Institute of Geochemistry, Chinese Academy of Sciences, Guangzhou 510640, China; e6.graham@qut.edu.au (E.G.); zhujx@gig.ac.cn (J.Z.); zhurl@gig.ac.cn (R.Z.); hehp@gig.ac.cn (H.H.); 3 School of Chemical and Environmental Engineering, China University of Mining and Technology, Beijing 100083, China; zhengsl@cumtb.edu.cn (S.Z.); zhimingsun@cumtb.edu.cn (Z.S.)

**Keywords:** metal-organic frameworks, diatomite, hydrogen, carbon dioxide, water vapor

## Abstract

Distinctive Cr-MOF@Da composites have been constructed using chromium-based metal-organic frameworks (MOFs) and diatomite (Da). The new materials have hierarchical pore structures containing micropores, mesopores and macropores. We have synthesized various morphologies of the MOF compound Cr-MIL-101 to combine with Da in a one-pot reaction step. These distinctive hierarchical pore networks within Cr-MIL-101@Da enable exceptional adsorptive performance for a range of molecules, including hydrogen (H_2_), carbon dioxide (CO_2_) and water (H_2_O) vapor. Selectivity for H_2_ or CO_2_ can be moderated by the morphology and composition of the Cr-MIL-101 included in the Cr-MOF@Da composite. The encapsulation and growth of Cr-MIL-101 within and on Da have resulted in excellent water retention as well as high thermal and hydrolytic stability. In some cases, Cr-MIL-101@Da composite materials have demonstrated increased thermal stability compared with that of Cr-MIL-101; for example, decomposition temperatures >340 ℃ can be achieved. Furthermore, these Cr-MIL-101@Da composites retain structural and morphological integrity after 60 cycles of repeated hydration/dehydration, and after storage for more than one year. These characteristics are difficult to achieve with many MOF materials, and thus suggest that MOF–mineral composites show high potential for practical gas storage and water vapor capture.

## 1. Introduction

Metal-organic frameworks (MOFs) are a class of crystalline materials with high potential for applications that require adsorption, separation, catalysis and drug delivery [1,2,3,4,5]. Adsorption-related applications, such as gas storage and water uptake, require a high specific surface area and well-defined pore networks in MOF materials, as shown for hydrogen (H_2_), carbon dioxide (CO_2_), methane (CH_4_) and water (H_2_O) vapor capture [6,7,8,9,10,11]. A recent review suggests that MOFs offer greater potential for H_2_ storage and re-use than existing technologies that are unlikely to meet the US Department of Energy’s (US DOE) 2020 guideline for a gravimetric storage capacity of 4.5 weight percent (wt.%) [12]. Other MOF compositions have been synthesized and evaluated for the capture and storage of CO_2_ with some success [13,14]. In these cases, a systematic approach to the design of a MOF for the adsorption of either H_2_ or CO_2_ often requires adjusting compositions of the metal, the ligand or the solvent to achieve an appropriate pore size and structure.

This synthesis strategy leads to the production of MOF compounds of substantially differing compositions. Replacing molecular building blocks (MBBs) such as metal ions and organic ligands is a common method to chemically tune MOFs [15,16]. However, this approach usually requires the utilization of different precursor chemistries, and leads to different types of products [16,17]. For example, the replacement of organic ligand BTDD (BTDD = bis(1H-1,2,3-triazolo (4¨C-b), (4′,5′-i)) dibenzo [1,4] dioxin) with BBTA (BBTA = 1H,5H-benzo (1,2-d), (4,5-d′)-bistriazole) demonstrates the tunability of pore size, but these two different products, defined as M_2_Cl_2_BTDD and M_2_Cl_2_BBTA, respectively, show different chemical compositions [16].

A preferred approach to MOF design is to target physisorption materials with a moderate or low heat of adsorption, in order to allow regeneration and re-use in a hydrolytic environment. For CO_2_ capture, as noted by Song et al., [13] this target would suggest a heat of adsorption below 60 kJ/mole. The utilization of controlled synthesis conditions with the same fundamental components, such as metal ion, organic ligand, solvent and counter-ion, has high potential use for the tunable production of key physical properties, such as thermal or hydrolytic stability, porosity, pore type and surface area [18,19,20]. For example, a strategy that targets square-shaped fluorine moieties, the Ni (II) ion and an inorganic substitute for (SiF_6_)^2−^, enabled the fine-tuning of hydrolytic stability for NdOFFIVE-1-Ni [21].

Chromium-based MOFs (Cr-MOFs), first synthesized and characterized by G. Ferey and co-workers [22,23], have been recognized as potential super adsorbents for a variety of gas molecules. The compound known as Cr-MIL-101, a Cr-MOF with the specific composition Cr_3_(F)(H_2_O)_2_O[(O_2_C)-C_6_H_4_-(CO_2_)]_3_·nH_2_O [22] (for comparison in this manuscript, the formula has been simplified as Cr_3_C_24_H_17_O_16_ = CrC_8_H_5.67_O_5.33_ omitting nH_2_O and using OH to replace F), shows good structural integrity as well as relatively high thermal and hydrolytic stability [24]. In this instance, Cr-MIL-101 was synthesized using HF, Cr(NO_3_)_3_∙9H_2_O, and the organic linking unit-terephthalic acid (H_2_BDC), followed by hydrothermal treatment at 220 °C for 6 h. Similar to Cr-MIL-101, many stable MOFs are constructed according to Pearson’s hard/soft acid/base principle, using hard bases including H_2_BDC, and high-valent metal ions including Cr^3+^ [25].

Nevertheless, the practical application of MOF materials encounters challenges due to poor chemical stabilities, low yields and potentially high manufacturing costs [26]. The open frameworks of MOFs built from the assembly of metal sites and organic linkers are vulnerable to water, which hinders their implementation in humid or water-associated environments [27]. Organic linkers are likely to decompose at high temperatures, which restricts their use under elevated temperature conditions. Additionally, the manufacturing costs are high due to low yields; for example, the yields for MIL-101 are only ~32–42% [28]. One proposed solution to overcome these obstacles is to build MOFs with a composite or substrate as a carrier [18,29,30]. Some silicate minerals could be promising due to the advantages of their abundance in nature and their chemical stability. In particular, diatomite (Da) has been used as a carrier to build MOF-based catalysts [31] and adsorbents [32]. It is well-acknowledged that porosity plays a decisive role in the adsorptive performance of porous materials [33], and, to date, MOFs have usually contained micro- and mesopores [34].

In this work, we have demonstrated that a diatomite (Da), used as a micron-scale three-dimensional building unit, can be successfully integrated with Cr-MIL-101 components. The Da with mainly macropores is compatible with Cr-MIL-101, which has mesopores and micropores. The resulting heterogeneous composite materials show three topologies—micro-, meso- and macroporous—as well as superior adsorptive performances for a range of molecules. In addition, the tunability of crystal morphologies, grain sizes and porosities that is characteristic of Cr-MIL-101 can result in the elicitation of useful physical properties via these composites.

## 2. Materials and Methods

### 2.1. Materials

Chromium (III) nitrate nonahydrate (Cr(NO_3_)_3_·9H_2_O, 99 wt.%), terephthalic acid (H_2_BDC, 98 wt.%), N,N-dimethylformamide (DMF, 99.8 wt.%), absolute ethanol (100 wt.%) and hydrochloric acid (HCl, 32 wt.%) were purchased from Sigma-Aldrich Co. (Sydney, Australia). All chemicals were used as received without further treatment. Diatomite was provided by the former Mount Sylvia Diatomite Pty Ltd. (now ChalkMine Australia) in Queensland, Australia. The diatomite is denoted as Da in this article. Deionized water with a resistivity of 18.2 MΩ·cm was obtained from a Milli-Q System (Millipore, Merck, Germany).

### 2.2. Synthesis of Cr-MIL-101 and Cr-MIL-101@Da

**Figure 1:**
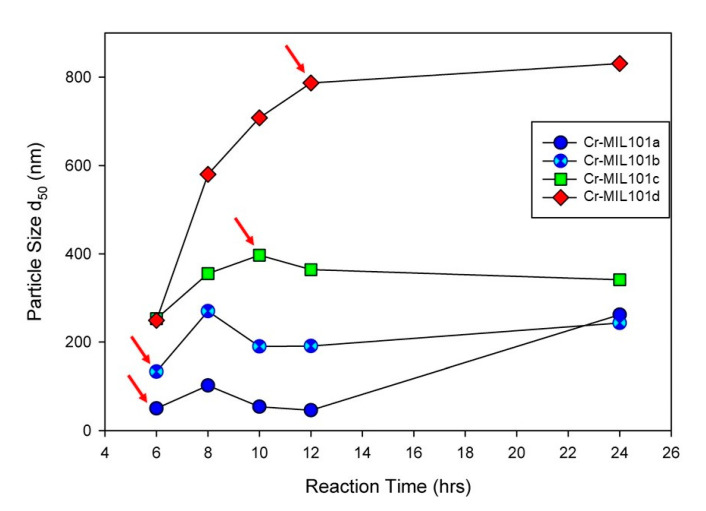
Particle size (d_50_) of Cr-MIL-101 synthesized under 1 mM to 4 mM precursor dosage with increasing reaction time from 6 h to 24 h, demonstrating tunability of grain sizes of Cr-MIL-101. Arrows refer to specific morphologies of Cr-MIL-101 combined with Da to form MOF-Da composites.

Modifications to a common procedure for synthesizing Cr-MIL-101 [22] have been used for this work. Firstly, the syntheses reported in this work did not use hydrofluoric acid (HF) as a mineralizing agent, which is beneficial to the environment. In addition, the hydrothermal reaction was undertaken at 180 °C for a range of time periods and metal ion/organic ligand concentrations (the molar ratio of metal ion to organic ligand = 1:1). For example, X mmol of Cr (NO_3_)_3_∙9H_2_O and H_2_BDC (X = 1–4) were stirred in 1060 mmol of Milli-Q water for 0.5 h. The mixture was then transferred to a 100 mL Teflon-lined autoclave and hydrothermally treated at 180 °C for Y h (Y = 6–24 h). After cooling to room temperature, all samples were recovered from suspensions by centrifugation, and washed with 20 mL of DMF twice at 80 °C for 1 h each to remove excess organic linker residues, and subsequently with 20 mL of absolute ethanol twice at 80 °C for 1 h each to remove residual DMF. The green crystalline powders were dried at 60 °C for 12 h, and then activated at 150 °C for 24 h. Four Cr-MIL-101 samples, denoted as Cr-MIL-101a, Cr-MIL-101b, Cr-MIL-101c and Cr-MIL-101d, respectively, were obtained at precursor concentrations of 1, 2, 3 and 4 mM. As identified in Figure 1, the reaction time for each Cr-MIL-101 sample used in this work (with arrows) was varied in order to obtain a specific MOF morphology or size.

**Figure 2:**
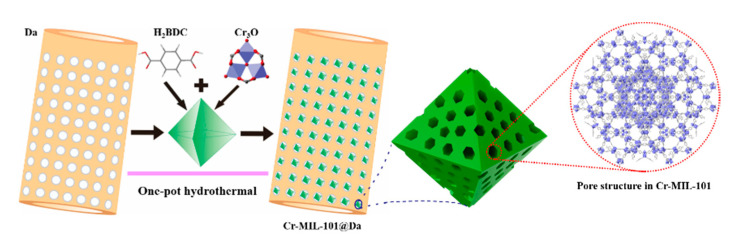
Schematic illustration of the construction of Cr-MIL-101@Da.

Similar to the synthesis of Cr-MIL-101, the general synthesis process for the composite material —Cr-MIL-101@Da is schematically illustrated in Figure 2. Precursor MOFs and diatomite (Da) were added simultaneously with products obtained via a one-pot hydrothermal treatment. Depending on the reaction conditions for Cr-MIL-101, four prototype Cr-MIL-101@Da materials with nano-sphere-, sphere-, tetrakaidecahedron- and octahedron-shaped Cr-MIL-101 particles on Da were produced. These composite products are referred to as Cr-MIL-101@Da-1, Cr-MIL-101@Da-2, Cr-MIL-101@Da-3 and Cr-MIL-101@Da-4, respectively. The products occurred as green powders and transitioned to a darker color from Cr-MIL-101@Da-1 to Cr-MIL-101@Da-4.

### 2.3. Bulk Properties

Powder X-ray diffraction (PXRD) patterns were collected using a SmartLab diffractometer (Cu Kα source, 40 kV and 40 mA, Rigaku, Tokyo, Japan) operating in Bragg–Brentano geometry with a Hypix 3000 detector. Patterns were acquired from 2θ of 2° to 75° at a step size of 0.01° 2θ and scan speed of 1.219° 2θ/min. Scanning electron microscopy (SEM) images and energy-dispersive X-ray spectroscopy (EDS) elemental maps were obtained with a field-emission scanning electron microscope (VP Zeiss Sigma, Berlin, Germany). Transmission electron microscopy (TEM) images were obtained with a JEM-2100 with a LaB_6_ filament (JEOL, Tokyo, Japan) and equipped with an Oxford Instruments SDD XMax 50 mm^2^ detector.

The particle size distributions of the products were determined in absolute ethanol using a Malvern ZetaSizer (Nano ZS90, Malvern, London, UK). Thermogravimetric analyses (TGA) to evaluate the thermal stabilities of MOFs were performed on samples pressed in Al_2_O_3_ holders and heated from ambient temperature to 1000 °C with a 10 °C/min ramp rate in a 50 mL/min N_2_ atmosphere using a Netzsch Jupiter instrument (STA449F3, Netzsch, Selb, Germany).

### 2.4. Chemical Analyses

The elemental compositions of C, H and O were determined by a CHNOS elemental analyzer (TruSpec Micro, LECO, Chicago, IL, USA). After digestion, the content of Cr was determined with an inductively coupled plasma optical emission spectrometer (ICP-OES, Perkin Elmer Optima 8300, Waltham, MA, USA). X-ray photoelectron spectroscopy (XPS) was obtained on an Axis Supra, Kratos photoelectron spectrometer (Axis Supra, Kratos, Tokyo, Japan) with a monochromatic Al Kα X-ray source (hν = 1486.6 eV). High-resolution XPS spectra were recorded with a pass energy of 20 eV. Binding energies were corrected using C 1s of 284.8 eV as the reference peak.

### 2.5. Gas Adsorption

Low-pressure gas adsorption experiments using nitrogen (N_2_), argon (Ar), hydrogen (H_2_) and carbon dioxide (CO_2_) were performed on a fully automated three-station gas sorption analyzer (3Flex, Micromeritics, Norcross, GA, USA). Samples were initially degassed at 150 °C for 6 h, and the weights of the gas-free samples were recorded upon degassing. The dosing manifold was purged several times with corresponding pure gas prior to the introduction of each gas analyte. Sample tubes were then mounted on the measurement station and a leakage test was conducted to ensure the accuracy of the measurement system. Upon completing the leakage test, the adsorption gas was introduced slowly with increments of gas pressure from vacuum (10 torr) to 1 bar, and the total weights of samples with the adsorbed gas were recorded. The equilibration interval was set at 45 s when the gas reached equilibrium pressure; the warmed free space was calculated after the measurement.

The N_2_ and Ar sorption–desorption isotherms were carried out at 77 K using liquid N_2_ and at 87 K using liquid Ar, respectively. BET specific surface area values were calculated on the basis of P/P_0_ = 0.05~0.25. The H_2_ storage experiments were operated at liquid N_2_ temperature (77 K) and at ambient temperature (298 K) by immersing the sample tube in a thermostatic liquid N_2_ or water bath. The CO_2_ uptake was obtained at 273.15 K and 293.15 K, respectively, by immersing the sample tube in a thermostatic water bath.

Water adsorption experiments were obtained in a programmable temperature and humidity test chamber (GDW-300, HASUC, Shanghai, China). Before tests were conducted, the weight of the totally dried holder without sample was recorded. Then 50 mg of each sample was added into the holder and dried at 150 °C for 2 h without a cover. The whole weight including the holder and sample was recorded and then it was transferred immediately into the sealed chamber to adsorb vapor. The weight changes at different time intervals were recorded, and the amount of vapor adsorbed was calculated via the following Equation (1):

(1) $$Mv\;=\;\frac{m_{t}-m_{1}}{m_{1}-m_{0}}\times 100\%$$

where *Mv* is the percentage of adsorbed water, *m_t_* is the total weight of sample and holder at time *t*, *m*_1_ is the total weight of sample and holder after drying and *m*_0_ is the weight of the dried holder. The water adsorption–desorption capacities were evaluated by alternately changing relative humidity (RH) at 98% to RH 33% at 45 °C for successive cycles.

### 2.6. Isosteric Heat of Adsorption for CO_2_

Initially, the relationship curves of ln*P* vs. CO_2_ uptake were obtained on the basis of adsorption isotherms of each sample. The data were then fitted with a Virial-type expression (Equation (2)):

(2) $$\mathrm{Ln}P\;=\;\;\mathrm{Ln}N+\;\frac{1}{T}\;\sum_{i\;=\;0}^{m}a_{i}N_{i}+\;\sum_{j\;=\;0}^{n}b_{j}N_{j}$$

where *P* is the adsorption pressure (torr), *N* (mmol/g) is the CO_2_ uptake at pressure *P*, *T* is the operational temperature at adsorption (273.15 K or 293.15 K), *a_i_* and *b_j_* are Virial coefficients, and *m* and *n* are the number of coefficients. *m* and *n* are optimized until the extra *a_i_* and *b_j_* are negligible to the whole value of the equation with R^2^ ≥ 0.999. Finally, *Q_st_* was calculated according to the Clausius–Clapeyron equation (Equation (3)):

(3) $$Q_{st}\;=\;\;\frac{\left(\mathrm{Ln}\;P_{2}-\;\mathrm{Ln}\;P_{1}\right)\times \left(-R\right)}{\frac{1}{T_{2}}-\;\frac{1}{T_{1}}}$$

where *Q_st_* is the isosteric heat of adsorption, *R* is the universal gas constant, and *P*_1_ and *P*_2_ are the adsorption pressures at *T*_1_ (273.15 K) and *T*_2_ (293.15 K), respectively.

## 3. Results

We briefly describe the different forms of Cr-MIL-101 used to produce composite forms of Cr-MIL-101@Da. A range of morphologies and particle sizes for Cr-MIL-101 can be developed with variations in synthesis conditions, such as the substitution of H_2_O for HF [24,35], or by incorporating adducts (e.g., amino acid glycine) to provide functionality [36]. For this work, a consistent synthesis method for Cr-MIL-101 has been used to minimize the influence of MOF synthesis conditions on MOF@Da composite performance.

### 3.1. Cr-MIL-101

The PXRD patterns indicate that the crystallization of Cr-MIL-101 occurs when the precursor concentration of Cr(NO_3_)_3_∙9H_2_O or H_2_BDC varies between 1 mM and 4 mM (the concentration ratio of Cr(NO_3_)_3_∙9H_2_O to H_2_BDC = 1:1) for reaction times from 6 h to 24 h. Examples of these PXRD patterns for different Cr-MIL-101 synthesis conditions are shown in Supplementary Materials (Figure S1). In all cases, the Cr-MIL-101 products show a space group symmetry of Fd$\bar{3}$m (No. 227) and cell dimensions consistent with those of MIL-101 (Cr) synthesized by G. Ferey and co-workers [22]. The chromium trimers and organic linkers build super-tetrahedral units, the ordered assembly of which results in a Cr-MIL-101 structure containing both microporous and mesoporous cages.

We show that progressive changes in particle size and morphology can be achieved for Cr-MIL-101 using the synthesis method described above. Figure S2 shows the morphological evolution of Cr-MIL-101 crystals with changes in reaction time and precursor concentration. Particle morphologies transform from dominantly spherical at ~6 h for all precursor concentrations to predominantly tetrakaidecahedron at ~10 h of reaction for all precursor concentrations. With additional reaction times—up to 24 h—the predominant morphology is octahedral for all precursor concentrations. Nevertheless, with the manipulation of reactant concentrations and time, all three morphologies with variable sizes can be synthesized as shown in Figure S2. In general, an increase in precursor concentration increases the final product’s particle size, while a longer reaction time also influences particle size, but to a lesser extent.

**Figure 3:**
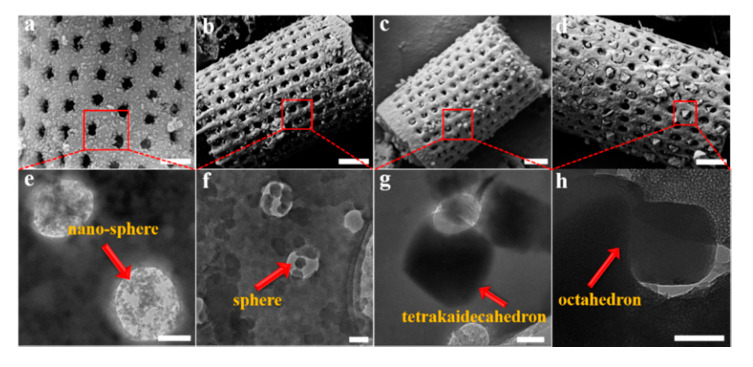
(**a**–**d**) SEM images of Cr-MIL-101@Da, scale bar = 1 μm, and (**e**–**h**) TEM images of the embedded Cr-MIL-101 particles in Da, scale bar = 200 nm.

The quantitative determination of particle size evolution with synthesis conditions is shown in Figure 1, which plots the average (d_50_) values for each reaction sequence. Data for each reaction, including the d_90_ and d_10_ particle sizes, are provided in the Supplementary Materials as Table S1. A notable feature of the data in Table S1 is the narrow size distribution (ranging from 65 nm to 223 nm; i.e., d_90_–d_10_) for all synthesis conditions. We have selected four examples of Cr-MIL-101 with specific particle sizes and morphologies for the construction of Cr-MIL-101@Da composites. These Cr-MIL-101 products are shown in Figure 3e–h (arrowed).

### 3.2. Cr-MIL-101@Da Composites

Da is an aggregation of the amorphous silica remnant shells, or frustules, of single-celled algae that have accumulated in lacustrine or marine sediments. These naturally occurring fossilized remains are usually highly symmetric in form, with low density and high macro-porosity, inert and insulating properties and high silica contents (SiO_2_·nH_2_O). These properties are not only useful for a wide range of industrial applications (e.g., filters, absorbents, soil amendments), but are suited to the formation of novel multi-porous composites. High-quality diatomite consists entirely of amorphous silica frustules, and hence an XRD pattern does not show reflections.

The XRD patterns of Cr-MIL-101@Da composites show reflections consistent with Cr-MIL-101, but with lower intensities compared to the MOF material, as shown in Supplementary Materials (Figure S3). These data show that the integrity of the Cr-MIL-101 structure is retained during the reaction to form a Cr-MIL-101@Da composite. For Cr-MIL-101@Da samples, SEM and TEM images reveal that the sphere-shaped Cr-MIL-101a and Cr-MIL-101b crystals with particle size smaller than the macropores of diatomite aggregate inside the pores and distribute on the surface of frustules (Figure 3a,b). However, the tetrakaidecahedron- and octahedron-shaped Cr-MIL-101c and Cr-MIL101d crystals are embedded in the holes of Da frustules (Figure 3c,d). In general, the dimensions of the Cr-MIL-101c and Cr-MIL-101d particles are larger than the macropores of Da, which may inhibit the growth of Cr-MIL-101 if they form inside the pores.

### 3.3. Chemistry

Bulk elemental analyses for Cr-MIL-101 samples are tabulated in detail in Table S2. These bulk analyses have been used to derive chemical formulae based on an estimation of oxygen content by conservation of charge balance. The formulae vary with synthesis conditions and the duration of the reaction between CrC_9.6_H_10.6_O_8_ (Cr-MIL-101a) and CrC_7.4_H_6.8_O_5.8_ (Cr-MIL-101d), which approximate the chemical formulae for Cr-MIL-101 (simplified as CrC_8_H_5.67_O_5.33_) initially synthesized by G. Ferey et al. [22], noting that the proportion of linker groups (e.g., C, O and H containing units) will vary with synthesis conditions. Consistent with this variation in chemistry, the powders display minor variations in color ranging from bottle green (Cr-MIL-101a) to reseda (Cr-MIL-101d). The analyses of Cr-MIL-101@Da samples demonstrate that the loadings of Cr-MIL-101 particles on Da vary from 16.90% to 28.92% (Table S2). The yield of Cr-MIL-101 reactions (based on chromium content) ranges between 39% and 49%. In comparison, the yield of the Cr-MIL-101@Da samples (based on the same method but including the weight of diatomite) ranges from 72% to 92%, indicating a relative increase of 33% to 43% (Table 1).

**Figure 4:**
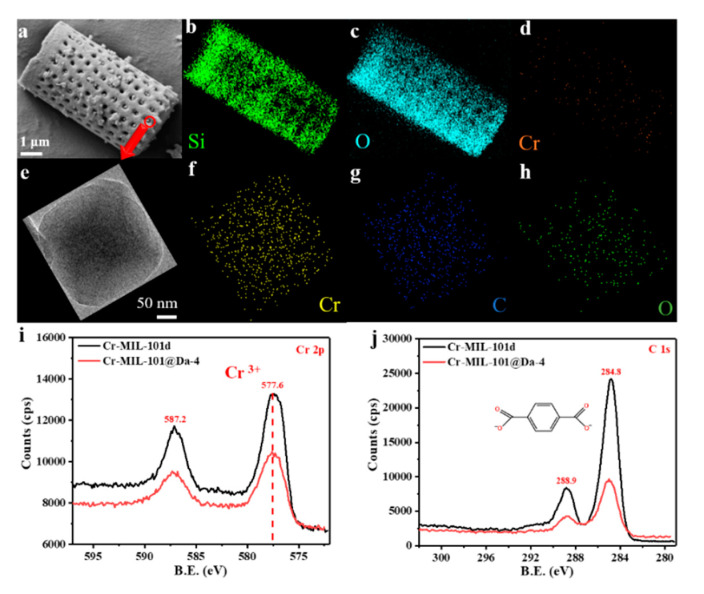
(**a**–**d**) SEM images and EDS elemental mapping analysis of Cr-MIL-101@Da-4, (**e**–**h**) TEM images and EDS elemental mapping analysis of the embedded Cr-MIL-101 particle in Da, (**i**,**j**) high-resolution XPS spectra of the Cr 2p, C 1s core level of Cr-MIL-101@Da-4 and Cr-MIL-101d.

The SEM and EDS elemental mapping of Si, O and Cr from a representative sample—Cr-MIL-101@Da-4—reflects a uniform distribution of Cr-MIL-101 particles on Da (Figure 4a–d). TEM-EDS analysis confirms that the embedded Cr-MIL-101 particle contains the elements Cr, C and O (Figure 4f–h). The wide scan surveys of XPS confirm the presence of carbon 1s and oxygen 1s states in all samples (Figure S4). The high-resolution XPS spectra show that the oxidation state of the chromium ions in the Cr-MIL-101 and Cr-MIL-101@Da-4 products synthesized in this work is trivalent (Figure 4i), and the charges of the cationic tri-nuclear clusters are balanced by COO^−^ anions (Figure 4j).

### 3.4. Porosity and Specific Surface Area

The nitrogen adsorption–desorption isotherms for all Cr-MIL-101 exhibit two stages of uptake, as shown in Figure S5a. The first N_2_ uptake occurs at low relative pressure with high adsorption, implying the presence of micropores. Argon adsorption–desorption isotherms for Cr-MIL-101 are also shown in Figure S5b. Compared with N_2_ adsorption, the rate of uptake of Ar by Cr-MIL-101 is significantly increased (note the difference in the vertical axis scale). The quadrupole moment and larger hard-sphere diameter of N_2_ at 3.681 Å compares with that for Ar at 3.148 Å. Table 2 lists the respective pore size diameters and BET specific surface area values for each product with the adsorption–desorption of N_2_ or Ar.

The N_2_ adsorption values increase in the series Cr-MIL-101a to Cr-MIL-101d consistently with the particle size variations shown in Figure 1. In contrast, the mesoporous pore diameter determined by N_2_ or Ar sorption decreases with the increasing particle sizes of all Cr-MIL-101, suggesting a more compact pore structure in larger particles. The BET specific surface areas measured using Ar for Cr-MIL-101 are larger than the values obtained using N_2_ (Table 2). The BET specific surface areas of Cr-MIL-101 range from 2191 m^2^/g to 2620 m^2^/g using Ar calculated for P/P_0_ = 0.05~0.25. The smaller nano-sized Cr-MIL-101a particles show the highest BET specific surface area, at 2620 m^2^/g.

**Figure 5:**
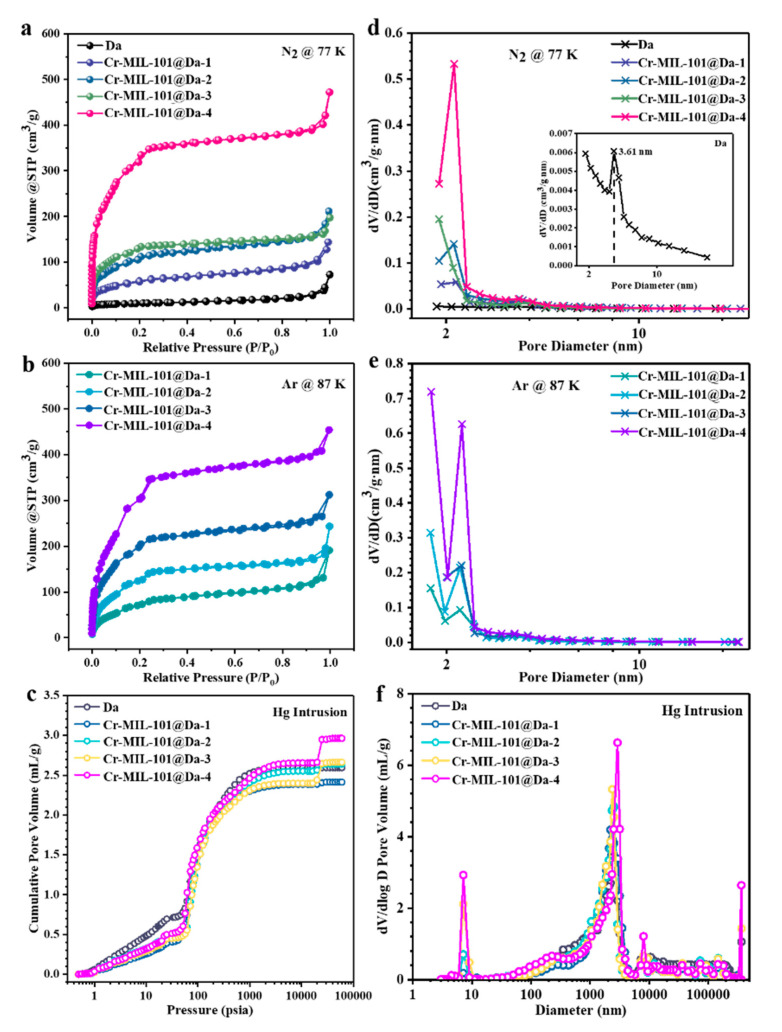
Porosity details for Da and Cr-MIL-101@Da. (**a**) N_2_ adsorption–desorption isotherms at 77 K, (**b**) Ar adsorption–desorption isotherms at 87 K, (**c**) cumulative intrusion curves measured by MIP, BJH desorption pore diameter distributions derived from (**d**) N_2_, and (**e**) Ar adsorption–desorption and (**f**) pore size distribution curves derived from MIP.

Da displays extremely low N_2_ adsorption capacity due to the lack of micro- and mesopores (Figure 5a). However, the N_2_ adsorption–desorption isotherms of Cr-MIL-101@Da exhibit two-step uptakes at low relative pressures, indicating the co-existence of two nanoporous windows (Figure 5a). The first uptake occurs at low relative pressure with especially high N_2_ adsorption, which implies the presence of micropores. Using Ar adsorption (Figure 5b), the BET specific surface areas of Cr-MIL-101@Da, except for Cr-MIL-101@Da-4, are larger than those measured using N_2_, confirming the presence of a higher proportion of micropores in Cr-MIL-101@Da (Table 2). The BET surface areas of the resultant Cr-MIL-101@Da materials extend to 1240 m^2^/g measured using N_2_ calculated on the basis of P/P_0_ = 0.05~0.25. These values are substantially higher than those determined for Da alone, at 37 m^2^/g. Additionally, the curve measured by mercury intrusion porosimetry (MIP) shows the presence of macropores in Da (Figure 5c). As expected, Cr-MIL-101@Da materials display a high cumulative Hg intrusion volume, confirming the existence of abundant macropores (Figure 5c).

The BJH desorption pore size distribution curves for N_2_ suggest that the mesopores of Cr-MIL-101@Da are predominantly at sizes of 2.9 nm and 3.4 nm, corresponding to the mesoporous cages in Cr-MIL-101 (Figure 5d) [22]. Interestingly, the BJH desorption pore size distribution curves of Ar verify the presence of micropores in Cr-MIL-101@Da, with an apparently high distribution of pore sizes smaller than 2.0 nm (Figure 5e). However, Da has abundant macropores, with a size distribution predominantly at 2.5 µm, attributed to the inside pore dimensions of cylindrical Da (Figure 5f). Notably, after the ordered assembly of Cr-MIL-101 particles into Da, the pore size distribution curves of Cr-MIL-101@Da are segmented, creating a range of macropores with peaks centered from 3.0 µm to 12.5 µm for Cr-MIL-101@Da (Figure 5f).

### 3.5. Gas Adsorption–Desorption

The data for three gases, H_2_, CO_2_ and H_2_O, are presented for Cr-MIL-101 products and the respective Cr-MIL-101@Da composites.

#### 3.5.1. Hydrogen Isotherms

H_2_ adsorption–desorption isotherms at 77 K and 298 K for Cr-MIL-101 particles are shown in Figure S6a,b, respectively. These isotherms show a differential uptake of H_2_, with Cr-MIL-101a being the most effective of the four Cr-MIL-101 products at both temperatures. At 77 K, both Cr-MIL-101c and Cr-MIL-101d show equivalent isotherms and similar responses to H_2_ uptake at 298 K. Cr-MIL-101b shows a relatively lower potential for H_2_ adsorption–desorption at 77 K compared with the other three Cr-MIL-101 products, and similarly at 298 K. For Cr-MIL-101a, the highest value for H_2_ uptake is 4.5 wt.% at 77 K and ~1 bar. This gravimetric uptake for H_2_ reduces to 0.07 wt.% at 298 K and ~1 bar.

**Figure 6:**
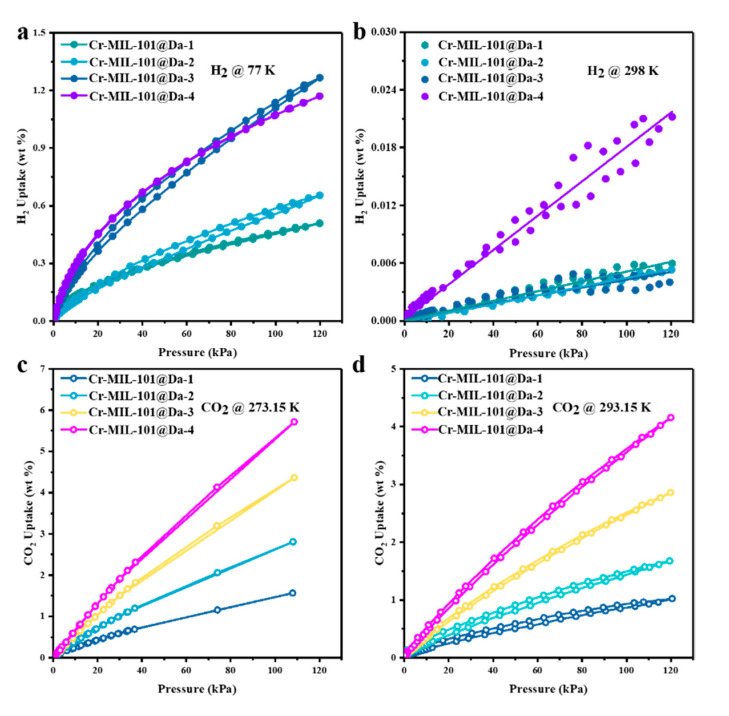
Gas adsorption performance of Cr-MIL-101@Da. (**a**,**b**) H_2_ adsorption–desorption isotherm at liquid N_2_ temperature and ambient temperatures and (**c**,**d**) CO_2_ adsorption–desorption isotherm at 273.15 K and 293.15K.

The H_2_ adsorption–desorption isotherms reveal high H_2_ uptake by Cr-MIL-101@Da, reaching a maximum of 13 mg H_2_ per gram (1.3 wt.%) of Cr-MIL-101@Da-3 at liquid N_2_ temperature and ~120 kPa (~1 bar) (Figure 6a). This value is comparable to many MOF materials in terms of H_2_ adsorption (Table S3). However, taking account of the 22.04 wt.% loading of Cr-MIL-101 on Da, these isotherm data are equivalent to 59 mg H_2_ per gram of Cr-MIL-101 (5.9 wt.%). For Cr-MIL-101@Da, this value for H_2_ gravimetric uptake is higher than reported for crystalline MOF materials under open low-pressure operational storage conditions (77 K and 1 bar), and higher than the US Department of Energy’s (DOE) target of 4.5 wt.% by 2020 [37]. The sorption sites of Cr-MIL-101@Da mainly result from Cr-MIL-101 particles, which have plenty of micropores and open metal sites or metal-building units [38,39]. The adsorption capacities of Cr-MIL-101@Da exhibit good correlation with the MOF contents in Da (Table S2). In addition, the variation in relevant Cr-MIL-101 particles also influences the adsorption properties of Cr-MIL-101@Da.

This study has evaluated the H_2_ storage abilities of these Cr-MOF@Da materials at ambient temperatures and low pressures. The adsorption isotherms display a linear increase in H_2_ uptake with absolute pressure (R^2^ ≥ 0.99), and achieve 0.022 wt.% for Cr-MIL-101@Da-4 at a pressure of about 1 bar (Figure 6b).

#### 3.5.2. Carbon Dioxide Isotherms

Contrary to the case for H_2_ adsorption–desorption, CO_2_ uptake is the lowest for Cr-MIL-101a at 293.15 K, and follows the morphology–particle size sequence to the highest value(s) for Cr-MIL-101d. At 273.15 K and 110 kPa, Cr-MIL-101b shows a marginally lower uptake of CO_2_, at 2.8 mmol/g, compared with Cr-MIL-101a at 3.0 mmol/g (Figure S6c). The highest observed uptake of CO_2_ for Cr-MIL-101d is 6.2 mmol/g at 273.15 K and 110 kPa.

Considering the successive hierarchical pore structures of Cr-MIL-101@Da, the adsorptive performance of Cr-MIL-101@Da for a larger gas molecule, such as CO_2_ (3.996 Å versus 2.968 Å for CO_2_ and H_2_, respectively), was also evaluated. Significantly, the CO_2_ adsorption–desorption isotherms indicate a maximum CO_2_ uptake of 5.7 wt.% (or 1.3 mmol/g) at 273.15 K and 1 bar for Cr-MIL-101@Da-4, which is equivalent to 19.7 wt.% (or 4.5 mmol/g) in terms of Cr-MIL-101 content (Figure 6c and Table S4). These values are among the highest for CO_2_ uptake for MOF and MOF-derived materials under low-pressure adsorption conditions [40,41,42] (Table S4). The adsorption capacities increase from Cr-MIL-101@Da-1 to Cr-MIL-101@Da-4, and this is attributed to the increased content of Cr-MIL-101 in Da. Therefore, the adsorption sites of Cr-MIL-101@Da for CO_2_ may come from the mesopores of Cr-MIL-101 particles [42]. Furthermore, the adsorption of CO_2_ by Cr-MIL-101@Da is an exothermic process, as inferred from the lower adsorption capacity at higher temperatures (i.e., ~293.15 K) (Figure 6d) compared with the capacity at ~273.15 K. The isometric adsorption heat (*Q_st_*) of CO_2_ for all of the Cr-MIL-101@Da composites are lower than 44 kJ/mol, which also indicates a physisorption between Cr-MIL-101@Da and CO_2_.

#### 3.5.3. Water Vapor Isotherms

H_2_O vapor adsorption–desorption values at 298 K, as wt.% adsorption at 98% RH and as desorption at 33% RH, are shown in Figure S7 for all four Cr-MIL-101 forms. Cr-MIL-101a and Cr-MIL-101d show the highest uptake of 214 wt.% and 201 wt.%, respectively, for H_2_O at 98% RH. Both Cr-MIL-101b and Cr-MIL-101c also have high values for uptake at ~173 wt.% H_2_O for 98% RH. With desorption at 33% RH, all four products retained significant proportions of water vapor between 34 wt.% and 83 wt.%.

**Figure 7:**
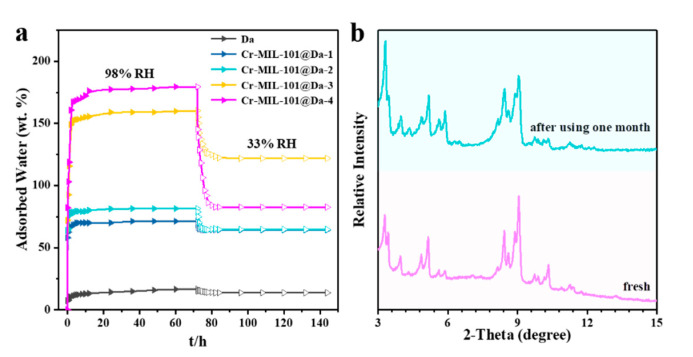
Water adsorptive performance and chemical stabilities. (**a**) water adsorption–desorption curve from relative humidity of 98% to 33% at 298 K, (**b**) comparison of XRD patterns between fresh and used Cr-MIL-101@Da-4 which was exposed with 98% RH–33% RH vapor pairs for over one month.

Da, as a macroporous silicate mineral, has a lower water adsorption capacity compared with Cr-MIL-101, showing only 16.8 wt.% water uptake at a relative humidity of 98% and 298 K (Figure S7). Interestingly, Da exhibits an excellent water retaining capacity upon contacting water because of the cylindrical structure of frustules, which appear to function as an effective macroporous container. As shown in Figure 7, after changing the relative humidity from 98% to 33%, Da desorbs only 2.95 wt.% of water. At a relative humidity of 98% and 298 K, our synthesized Cr-MIL-101@Da composite adsorbs 179.5 wt.% water vapor. The Cr-MIL-101@Da composites desorb less water when the relative humidity decreases from 98% to 33%, compared to Cr-MIL-101 materials (Figure 7 and Figure S7). The construction of hierarchical pore structures, including many newly generated successive pores, appears to favor an improvement of the water retaining capacity.

### 3.6. Thermal and Hydrolytic Stability

**Figure 8:**
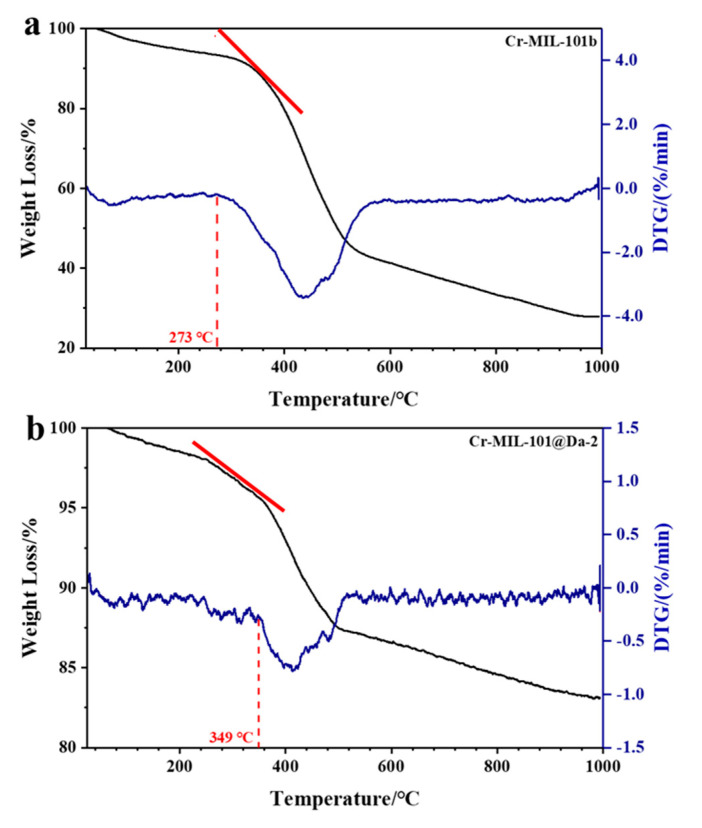
TG-DTG curves of (**a**) Cr-MIL-101b and (**b**) Cr-MIL-101@Da-2. Red tangent lines highlight the respective slope(s) and different weight loss rates for MOF-Da composites.

Compared with Cr-MIL-101, the Cr-MIL-101@Da composites show enhanced thermal stabilities. The TG-DTG curves reveal that the structural decomposition of all Cr-MIL-101@Da composites begins at higher temperatures than the corresponding Cr-MIL-101. Representative TG and DTG plots for Cr-MIL-101b and Cr-MIL-101@Da-2 are provided in Figure 8a,b. The decomposition of Cr-MIL-101 can be determined by variable temperature PXRD and by TG-DTG analysis. Variable temperature PXRD plots displayed at 100 °C intervals up to 400 °C (Supplementary Materials Figure S8) show that the four Cr-MIL-101 materials lose structural integrity between 200 °C and 350 °C, with the complete absence of structure by 350–400 °C. These PXRD data are consistent with the TG-DTG plots (Figures S8 and S9) that also identify the start of substantial weight loss. Compared with Cr-MIL-101 materials, Cr-MIL-101@Da materials show slower weight loss in the same temperature range (e.g., refer to red tangent lines in Figure 8), revealing an improved thermal stability. Estimated decomposition temperatures are compiled in Table 3 along with the decomposition temperatures for the precursor Cr-MIL-101 and Cr-MIL-101@Da materials.

The hydrolytic stability of Cr-MIL-101@Da is demonstrated by PXRD patterns before and after alternate exposure to 98% RH and 33% RH water vapor cycling every 12 h at 318 K for over one month. Figure 7b shows the “before” and “after” PXRD patterns for Cr-MIL-101@Da-4, which show a good retention of structure after 60 cycles of variable RH at 318 K. Furthermore, all of the synthesized Cr-MIL-101@Da samples demonstrate excellent hydrolytic stability (Figure S10).

## 4. Discussion

In general, research trends, particularly for Cr-based MOFs, have focused on (i) the syntheses of smaller particle-size products while retaining porosity benefits such as with Cr-MIL-101 [28,35,43], (ii) incorporating adducts, such as NH_2_ [44] or amino acid glycine [36], to the structure to provide specific functionality, or (iii) the utilization of “look alike” linkers to exploit the beneficial properties of Cr-organic structures, as in Cr-soc-MOF-1 [43]. For example, Towsif Adtab et al. [43] used a solvothermal reaction with tetratopic ligands and iron chloride to produce Fe-soc-MOF-1 as a precursor to enable trans-metalation to form Cr-soc-MOF-1. This compound shows high H_2_O uptake, achieving 1.95 g/g uptake of water vapor at 70% RH [43].

The attempt to produce a nano-scale particle size for Cr-MIL-101 products led to syntheses that did not require the use of HF [24,28,35], as in this study, as well as the exploration of other synthesis variables, such as water concentration and pH [24], and the use of alternative monocarboxylic acids as mediators [45]. More recently, acetic acid has been shown to be beneficial for the synthesis of a Cr-MIL-101 product with an average diameter of 90 nm, a high BET surface area and good yield (60%–75%) [35]. One of many rationales for the synthesis of nano-scale MOFs is the retention of bulk phase physical/chemical properties, with concomitant improvements in specific properties such as lower heats of adsorption, as shown for the nano-scale Zn-based compound MOF-5 [46].

Currently, the advantage and flexibility of MOF designs rely upon a ‘building block’ approach via combining two major components; for example, various metal oxide clusters (nodes) and multifunctional organic linkers (struts). However, with two major components there is limited flexibility to design for higher performance, and thus this becomes an obstacle to optimum fabrication for specific applications at a reasonable cost. In the last decade, MOF composite materials with polymer, graphene oxide or carbon nanotubes have been investigated for improved performance, with some success [47]. More recently, montmorillonite and attapulgite have also been evaluated as components of MOF composites. For example, montmorillonite influences the size of Zn-BDC crystals [48] and attapulgite is shown to selectively coordinate with metal ions in Cu-BTC to enable stability under thermal and hydrolytic variation [49]. In this work, we show that diatomite is an environmentally benign mineral with a three-dimensional macroporous morphology well suited to the formation of composites with MOFs.

### 4.1. Cr-MIL-101@Da Composites

The four pristine Cr-MIL-101 products from these syntheses share the same crystal structure with different preferred orientations, observed from the intensities of the same lattice planes and distinctive morphologies. The previous literature [23] shows that the base units in a Cr-MIL-101 structure are super-tetrahedral building blocks built from six organic linkers and four inorganic trimers. In our work, inorganic trimer building blocks are made from the assembly of three chromium octahedra sharing a μ_3_O atom, four oxygen atoms derived from dicarboxylates, and one oxygen atom from terminal water, instead of from a fluorine group when using the HF method. The construction between the super-tetrahedral units affords a well-defined porosity to Cr-MIL-101 containing both pentagonal and hexagonal channels.

Based on the characterization results, we have summarized the crystal growth mechanism for Cr-MIL-101 (see Figure S11). At the initial stage, granules nucleate with a spherical morphology, which has the most uniform surface energy. When the crystal nucleus starts to grow with reaction time, the {100} facet has a faster growth rate than that of the {111} facet (v{100} > v{111}) due to the higher surface energy of {100} based on a surface energy-driven mechanism, leading to the formation of a tetrakaidecahedron morphology [50]. According to the BFDH (Bravais, Friedel, Donnay and Harker) law, the slowest growth facet dominates the morphology of a crystal. Thus, the {100} facet disappears gradually, and the tetrakaidecahedral shape finally transforms into an octahedral shape due to growth of the {111} facet. On the other hand, the grain sizes of Cr-MOF crystals grow larger with the increasing reaction time and precursor dosage. The growth striations on Cr-MIL-101 observed using electron microscopy are a direct proof of the proposed mechanism. The growth striations of standard tetrakaidecahedrons and octahedrons are in a triangle and hexagonal format, respectively. However, the observed growth striations of the hexagonal format are almost spherical, which also supports the evolution of Cr-MOF from a sphere to a tetrakaidecahedron, and then to an octahedron.

Comparable to the size and shape of the corresponding pristine Cr-MIL-101 particles, in Cr-MIL-101@Da-1, nanoscale Cr-MOF particles mainly aggregate in the holes and distribute on the surface of Da. In Cr-MIL-101@Da-2, the sphere-shaped Cr-MOF particles mainly aggregate in the holes and distribute on the surface of Da. In Cr-MIL-101@Da-3, the tetrakaidecahedron-shaped Cr-MOF particles are mainly embedded in Da. In Cr-MIL-101@Da-4, the octahedron-shaped Cr-MOF particles are mainly embedded in Da.

The binding mechanism between Da and Cr-MIL-101 is probably due to electrostatic interactions. At the initial reaction pH of around 2.3, Da is negatively charged, which may attract positively charged Cr-MIL-101. The zeta potentials of all Cr-MIL-101@Da are located between those of Da and Cr-MIL-101 (Figure S12). It is also possible that metal ions (such as Cr^3+^) may be attracted to the surface of Da, then react with H_2_BDC in situ to form Cr-MIL-101 particles on the surface(s) of Da. Chemical bonds may form between the MOF metal ions and surface hydroxyl groups of Da in a similar way as was reported for Cu-BTC and attapulgite [49]. We also infer that Da may induce MOF crystal growth during synthesis, similar to our nano zero-valent iron (nZVI), which can be induced by Da to form well-dispersed individual spherical particles rather than aggregates of fibers, as observed in pristine nZVI [51].

### 4.2. H_2_ and CO_2_ Adsorptive Performance

We tabulated data for a wide range of MOF materials in Table S3 of the Supplementary Materials. Most MOF materials display H_2_ adsorption capacities in the range of 1–2 wt.%, and the highest published H_2_ uptake in Table S3 is 4.5 wt.%, obtained by MOF-5 at 77 K and ~1 bar. Nano-scale Cr-MIL-101a displays the highest H_2_ uptake among the four types of Cr-MIL-101 (Figure S6a,b), which results from a higher proportion of micropores in Cr-MIL-101a. The larger Cr-MIL-101c and Cr-MIL-101d samples, with relatively large specific surface areas and high porosities, show the next highest H_2_ uptake, followed by Cr-MIL-101b.

The ultimate target for storage materials is to achieve H_2_ storage at an ambient temperature and at a pressure suitable for practical applications [39,52]. Nevertheless, H_2_ storage at ambient temperatures is still a challenge for many materials evaluated to date, and an appropriate design for H_2_ storage at ambient temperatures has not yet been achieved. The adsorption isotherms of Cr-MIL-101 at ambient temperatures display a linear increase in H_2_ uptake with absolute pressure, and reach 0.07 wt.% for Cr-MIL-101a at about 1 bar (Figure S6b). On the basis of a linear trajectory estimate, the H_2_ uptake for Cr-MIL-101a could reach ~7.0 wt.% at 100 bar and 298 K. This value for H_2_ uptake is much higher (a 55.6% increase) than the Department of Energy’s (DOE) target of 4.5 wt.% for 2020 [37], implying good potential for H_2_ storage. For Cr-MIL-101@Da materials, the adsorption isotherms display a linear increase in H_2_ uptake with absolute pressure (R^2^ ≥ 0.99), and reach 0.022 wt.% for Cr-MIL-101@Da-4 at a pressure of about 1 bar (Figure 6b). Based on a linear trend estimate, the H_2_ uptake for Cr-MIL-101@Da-4 could reach 2.2 wt.% at 100 bar and 298 K. This value suggests high potential for ambient H_2_ storage at pressure [12], considering the proportionally low percentage of Cr-MIL-101 in these composites.

Octahedral-shaped Cr-MIL-101d, with the highest N_2_ uptake and largest particle size, exhibits the highest CO_2_ uptake (Figure S6c), while the nano-scaled Cr-MIL-101a displays the highest H_2_ uptake (Figure S6a,b), suggesting that micropores and mesopores, respectively, dominate the adsorption processes for H_2_ and CO_2_ in these MOF compounds

These results reflect the significant and useful role of variable pore structures for specific applications, and exemplify tunable products by targeted synthesis. Increased temperature results in a decrease in CO_2_ capture for Cr-MIL-101, as observed in Figure S13 (Supplementary Materials), which shows a comparison between results at 273.15 K and 293.15 K. The heat of adsorption for CO_2_ suggests a physical interaction between CO_2_ and Cr-MIL-101 particles and Cr-MIL-101@Da materials, since all the values are lower than 44 kJ/mol (Figures S6d and S14).

### 4.3. H_2_O Adsorptive Performance

MOF materials with typical hydrophilic metal sites are reported to have potential for water-related applications, such as water harvesting in arid areas, sea water desalination and adsorption heat pumps (AHP), due to their superior water adsorption capacities [16,53,54]. As mentioned (Figure S7), at relative humidities of 98% (298 K) and 33% (298 K), synthesized Cr-MIL-101@Da-4 adsorbs 179.5 wt.% and 82.6 wt.% water vapor, respectively, while Cr-MIL-101@Da-3 adsorbs 160.3 wt.% and 121.8 wt.% water vapor, respectively. These values outperform the recently recorded Ni_2_Br_2_BTDD (64 wt.% at RH below 25%) [55], and is comparable to that of pure Cr-soc-MOF-1, the highest water adsorbent (195 wt.%) reported so far [43]. The exceptional water adsorption and retention capacity of Cr-MIL-101@Da may enable effective water harvesting from extremely arid regions. In addition, Cr-MIL-101b and Cr-MIL-101c show lower adsorption capacities for water than Cr-MIL-101a and Cr-MIL-101d, demonstrating the combinative effect of porosity and specific surface area on water adsorption.

The adsorption capacities of Cr-MIL-101@Da can be controlled and recyclable, through appropriate combinations of the structure, morphology, grain size and loading amounts of Cr-MIL-101 crystals on Da, enabled by the range of adsorptive performances of these four synthesized Cr-MIL-101@Da materials. Significantly, Cr-MIL-101@Da’s hierarchical structures show high water-retaining capacities when measured over adsorption–desorption cycles of relative humidity. This capacity is a unique feature that is rarely observed in other water adsorbents utilizing MOF materials. We ascribe this to successive hierarchical pore networks and cylindrical structures of Cr-MIL-101@Da, which temporarily “lock” water molecules in the pore structures.

Based on discussions from Section 4.2 and Section 4.3, it is obvious that our composite materials’ high adsorptive performances are due to the synergistic effects of Da and Cr-MIL-101. This is because, if they are just physically mixed, only pristine MOF in the mixture will contribute to the adsorption, as Da only has very low adsorption capacities.

### 4.4. Thermal and Hydrolytic Stability

Our materials show high thermal stabilities, as observed from the thermogravimetric and differential thermogravimetry (TG-DTG) curves (Figure 8 and Figure S9), which are higher than the value of 275 °C reported for conventional Cr-MIL-101 [24]. The synthesized materials also exhibit good hydrolytic stabilities, as demonstrated by the unaltered structures of Cr-MIL-101@Da after being subjected to 98% RH and 33% RH vapor cycling every 12 h at 318 K for over one month (Figure 7b and Figure S10). The SEM images in Figure S15 demonstrate that the Cr-MIL-101 particles are still well dispersed in diatomite after two months. We have also tested the long-term stabilities (now > 20 months, with ongoing testing) of some selected Cr-MIL-101@Da samples after they have been stored at atmosphere in glass sample vials only, with push-on caps without any additional seal, protective gas or vacuum. The XRD analysis of these long-term stored samples under ambient conditions demonstrates that these composites still display high crystallinities, and suggests exceptional storage stability.

This enhancement of stabilities for MOF crystalline materials may enable industrial applications, such as with adsorption-heat pumps (AHP), solar-thermal systems and catalysis. In addition, the yields of the Cr-MIL-101@Da materials with similar adsorption performance and higher thermal stability are dramatically increased compared with those of Cr-MIL-101 alone, with an increase between 33% and 43%, as shown in Table 1. This attribute may significantly improve the cost to benefit ratio typically ascribed to MOF materials and other adsorbents for practical applications at an industrial scale.

## 5. Conclusions

We have constructed a novel Cr-MIL-101@Da material using an HF-free hydrothermal method, which enables the formation of hierarchical structures containing micropores, mesopores and macropores that cooperatively benefit adsorption/desorption, as well as hydrolytic and thermal performance. These distinctive hierarchical structures exhibit excellent adsorption for H_2_ (5.9 wt.% in terms of Cr-MOF content at 77 K and pressure ~1 bar), CO_2_ (5.7 wt.% at 273.15 K and ~1 bar) and water vapor (179 wt.% at 298 K and RH = 98%). Using four morphological variants of Cr-MIL-101 with specific adsorption capacities for H_2_ and CO_2_, we have constructed composites with diatomite that demonstrate a wide range of properties, reflecting the cooperative influence(s) of MOF and diatomite. The pore size distribution in these composites enables high water retention capacity and improved thermal stability compared with Cr-MIL-101 alone. In addition, all four composites undergo water vapor adsorption–desorption cycles without loss of structural integrity for up to a month. These Cr-MIL-101@Da composites also retain structural integrity after ambient storage of more than 20 months. The extension of these outcomes and the further application of these fundamental concepts to other types of MOF–mineral composites are likely to prove fruitful areas of research.

## Figures and Tables

**Table 1:** Comparison of the yields of Cr-MOF and Cr-MOF@Da

Samples	Yield (%)	Samples	Yield (%)	Increased Yield (%)
Cr-MIL-101a	49	Cr-MIL-101@Da-1	92	43
Cr-MIL-101b	36	Cr-MIL-101@Da-2	79	43
Cr-MIL-101c	36	Cr-MIL-101@Da-3	75	39
Cr-MIL-101d	39	Cr-MIL-101@Da-4	72	33

**Table 2:** Adsorption–desorption parameters measured for Cr-MIL-101 and Cr-MIL-101@Da.

	BET Surface Area (m^2^/g)		Mesoporous Pore Diameter ^a^		Microporous Pore Diameter ^b^		Total Pore Volume (cm^3^/g) ^c^	
	N_2_	Ar	N_2_	Ar	N_2_	Ar	N_2_	Ar
Cr-MIL-101a	1395	2621	6.9	5.6	0.68	0.81	0.95	1.65
Cr-MIL-101@Da-1	218	262	6.1	3	0.72	0.81	0.18	0.17
Cr-MIL-101b	1535	2406	5.3	3.5	0.69	0.75	0.69	1.01
Cr-MIL-101@Da-2	415	458	4	2.7	0.75	0.8	0.16	0.21
Cr-MIL-101c	1797	2552	2.6	2.3	0.72	0.79	0.44	0.67
Cr-MIL-101@Da-3	482	680	3	2.9	0.71	0.75	0.12	0.25
Cr-MIL-101d	2391	2191	2.3	2.3	0.71	0.75	0.53	0.5
Cr-MIL-101@Da-4	1241	1146	2.9	2.6	0.75	0.79	0.3	0.37
Da	37	NA	8.5	NA	0.73	NA	0.05	NA

^a^—BJH desorption average pore diameter (4V/A), unit: nm. ^b^—Horvath–Kawazoe median pore width, unit: nm. ^c^—BJH adsorption cumulative volume of pores between 1.7 nm and 300.0 nm diameter.

**Table 3:** Decomposition temperatures for Cr-MIL-101 and Cr-MIL-101@Da.

Sample	MOF	MOF@Da	ΔT_d_ (°C)	Ref
	T_d_ (°C)	T_d_ (°C)		
Cr-MIL-101a	330	341	11	This work
Cr-MIL-101b	273	349	76	This work
Cr-MIL-101c	281	341	60	This work
Cr-MIL-101d	276	276, 305	0, 29	This work
MIL-101	275	-	-	[24]

## Supplementary Materials

The following are available online at https://www.mdpi.com/1996-1944/13/21/4700/s1, Figure S1: The calculated (green lines), observed (dark lines) and difference (grey lines) PXRD profiles as well as the allowed reflections (blue lines) of Cr-MIL-101 compounds fitted using Rietveld method (a) Cr-MIL-101-a, (b) Cr-MIL-101-b, (c) Cr-MIL-101-c and (d) Cr-MIL-101-d, Figure S2: SEM images of Cr-MIL-101 synthesized under 1 mM to 4 mM precursor dosage with increasing reaction time from 6 h to 24 h, demonstrating tunability of morphologies of Cr-MIL-101. Scale bars = 500 nm, Figure S3: PXRD patterns of Cr-MIL-101@Da-1, Cr-MIL-101@Da-2, Cr-MIL-101 @Da-3 and Cr-MIL-101-@Da-4, respectively, Figure S4: The wide survey scans of Cr-MIL-101@Da-1, Cr-MIL-101@Da-2, Cr-MIL-101 @Da-3 and Cr-MIL-101-@Da-4, respectively, Figure S5: (a) N_2_ (77 K) and (b) Ar (87 K) adsorption–desorption isotherms for Cr-MIL-101 samples, demonstrating tunability of porosities of Cr-MIL-101, Figure S6: Adsorptive performance of Cr-MOFs. (a,b) H_2_ sorption–desorption isotherms at liquid nitrogen (77 K) and ambient (298 K) temperatures, (c) CO_2_ sorption–desorption isotherms at 273.15 K, (d) CO_2_ adsorption enthalpies, (e) water adsorption–desorption curves from relative humidity of 98% to 33% at 298 K and (f) comparison of XRD patterns between fresh and used Cr-MIL-101a exposed with 98% RH–33% RH vapor pairs for over one month, Figure S7: Water adsorption and retaining capacities of various adsorbents from RH 98 % to 33% at 298 K, Figure S8: VTPXRD patterns of (a) the decomposition of Cr-MIL-101a occurs between 300 and 350 °C, (b) the decomposition of Cr-MIL-101b occurs between 200 and 300 °C, (c) the decomposition of Cr-MIL-101c occurs between 300 and 350 °C and (d) the decomposition of Cr-MIL-101d occurs between 200 and 300 °C, Figure S9: TG-DTG curves of (a) left: Cr-MIL-101a and right: Cr-MIL-101@Da-1, (b) left: Cr-MIL-101c and right: Cr-MIL-101@Da-3, (c) left: Cr-MIL-101d and right: Cr-MIL-101@Da-4, Figure S10: Comparison of XRD patterns between fresh Cr-MIL-101@Da (black) and Cr-MIL-101@Da exposed to 98% RH–33% RH vapor pairs for one month (red) for (a) Cr-MIL-101@Da-1, (b) Cr-MIL-101@Da-2 and (c) Cr-MIL-101@Da-3, respectively, Figure S11: Transformation mechanism for Cr-MIL-101, Figure S12: Zeta potentials of Da, Cr-MIL-101 and Cr-MIL-101@Da at various solution pH, Figure S13: The gravimetric adsorption capacities of Cr-MIL-101 for CO_2_ at (a) 273.15 K and (b) 293.15 K, Figure S14: CO_2_ adsorption–desorption isotherms (left) and isometric adsorption heat (*Q_st_*) of CO_2_ calculated from the corresponding adsorption isotherms (right) for (a) Cr-MIL-101@Da-1, (b) Cr-MIL-101@Da-2, (c) Cr-MIL-101@Da-3 and (d) Cr-MIL-101@Da-4, Figure S15: SEM images of Cr-MIL-101@Da-3 after using for two months, Table S1: Particle size distribution of Cr-MIL-101, Table S2: Elemental compositions, formulae and MOF contents in Da, Table S3: Total H_2_ uptake of the obtained Cr-MIL-101@Da hierarchical structures in comparison with the best MOF materials reported so far, Table S4: Total CO_2_ uptake of the obtained Cr-MIL-101@Da materials in comparison with the best MOF materials reported so far.
