# Supplementary material for: Diatomite-Metal-Organic Framework Composite with Hierarchical Pore Structures for Adsorption/Desorption of Hydrogen, Carbon Dioxide and Water Vapor
Source: Materials (Basel). 2020 Oct 22;13(21):4700. doi: 10.3390/ma13214700 (PMC7659967; doi:10.3390/ma13214700)
Supplement: Supplementary file 1 [file materials-13-04700-s001.pdf]

materials

MDPI

Supplementary Materials

Diatomite-Metal-Organic Framework Composite with Hierarchical Pore Structures for Adsorption/Desorption of Hydrogen, Carbon Dioxide and Water Vapor

Gaofeng Wang [1,2], Elizabeth Graham [2], Shuilin Zheng [3], Jianxi Zhu [2], Runliang Zhu [2], Hongping He [2], Zhiming Sun [3], Ian D. R. Mackinnon [1] and Yunfei Xi [1,*]

1 Institute for Future Environments and Science and Engineering Faculty, Queensland University of Technology (QUT), Brisbane, Queensland 4001, Australia; wanggaofeng@gig.ac.cn (G.W.); ian.mackinnon@qut.edu.au (I.D.R.M.)
2 CAS Key Laboratory of Mineralogy and Metallogeny, Guangdong Provincial Key Laboratory of Mineral Physics and Material Research and Development, Guangzhou Institute of Geochemistry, Chinese Academy of Sciences, Guangzhou 510640, China; e6.graham@qut.edu.au (E.G.); zhujx@gig.ac.cn (J.Z.); zhurl@gig.ac.cn (R.Z.); hehp@gig.ac.cn (H.H.)
3 School of Chemical and Environmental Engineering, China University of Mining and Technology, Beijing 100083, China; zhengsl@cumtb.edu.cn (S.Z.); zhimingsun@cumtb.edu.cn (Z.S.)
* Correspondence: y.xi@qut.edu.au; Tel.: +61-07-3138-1995

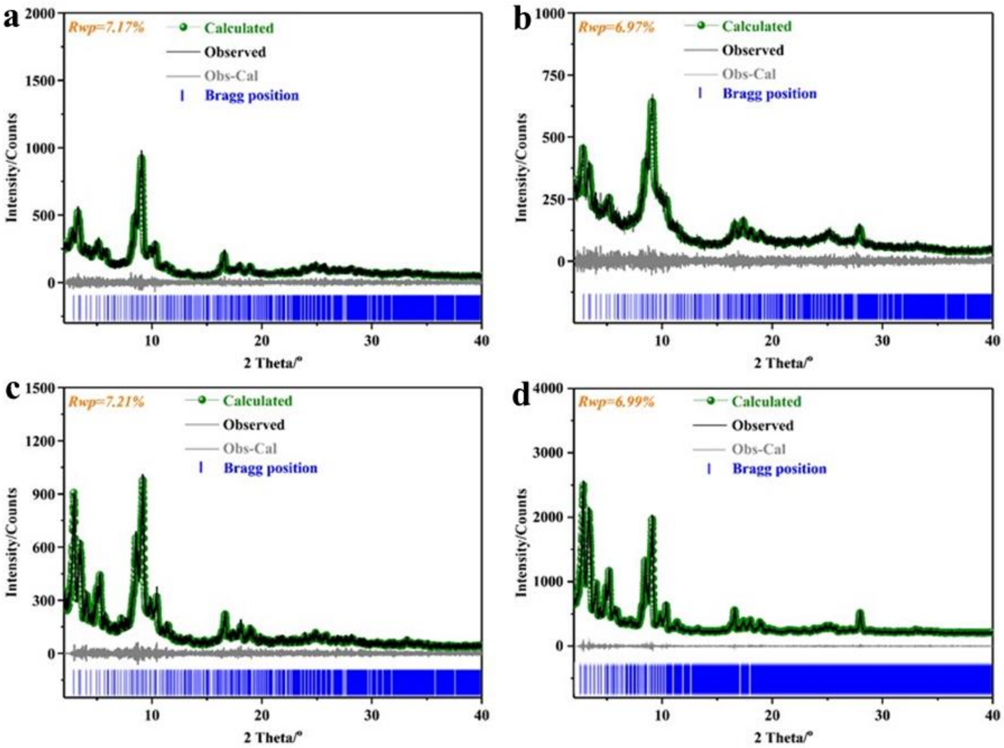

Figure S1. The calculated (green lines), observed (dark lines) and difference (grey lines) PXRD profiles as well as the allowed reflections (blue lines) of Cr-MIL-101 compounds fitted using Rietveld method (**a**) Cr-MIL-101-a, (**b**) Cr-MIL-101-b, (**c**) Cr-MIL-101-c and (**d**) Cr-MIL-101-d.

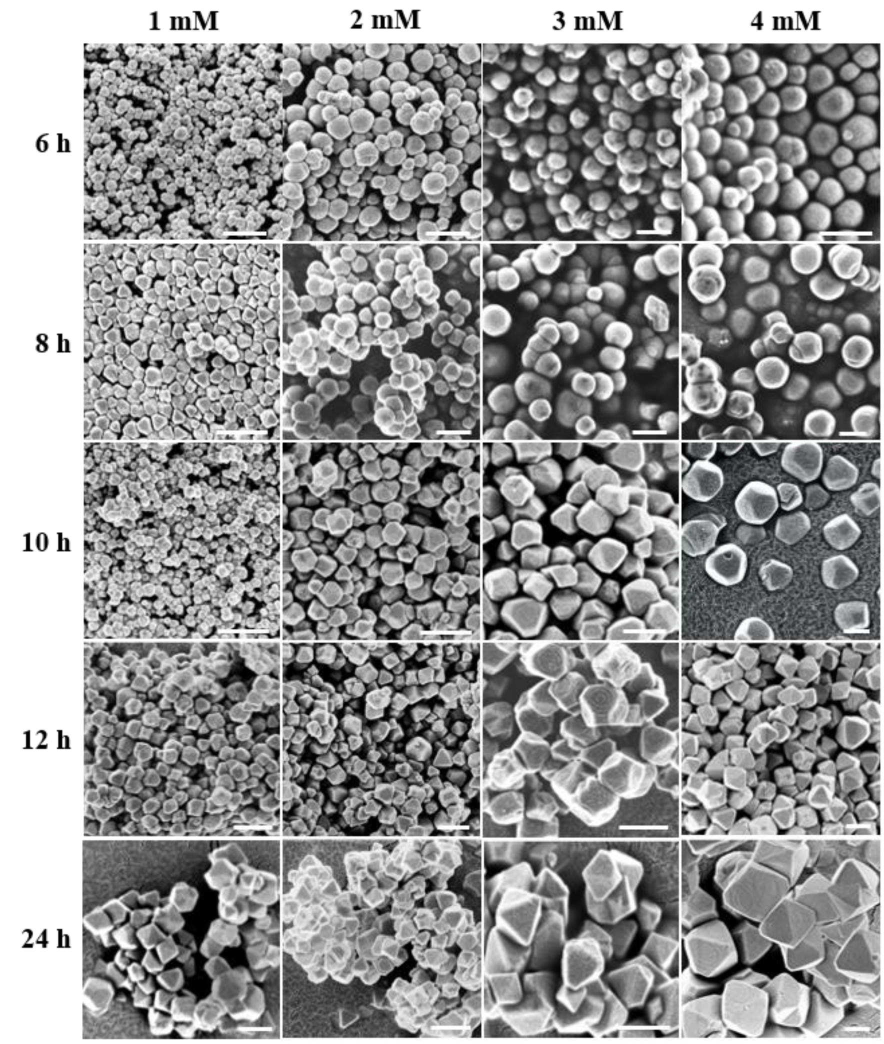

Figure S2. SEM images of Cr-MIL-101 synthesized under 1 mM to 4 mM precursor dosage with increasing reaction time from 6 h to 24 h, demonstrating tunability on morphologies of Cr-MIL-101. Scale bars = 500 nm.

Table S1. Particle size distribution of Cr-MIL-101.

Samples	Reaction time/h	Particle size distribution		
		d_{10}/nm	d_{50}/nm	d_{90}/nm
Cr-MIL-101a	6	50	67	80
	8	102	153	208
	10	54	73	98
	12	46	64	85
	24	262	318	369
Cr-MIL-101b	6	96	133	181
	8	242	270	295
	10	143	190	255
	12	132	191	291
	24	165	243	369
Cr-MIL-101c	6	169	253	373
	8	281	355	456
	10	296	397	533
	12	301	364	450
	24	265	341	449
Cr-MIL-101d	6	207	249	257
	8	452	580	687
	10	583	708	835
	12	646	787	879
	24	685	831	1031

Table S2. Elemental compositions, formulae and MOF contents in Da.

Samples	Cr (wt. %)	C (wt. %)	H (wt. %)	Formula
Cr-MIL-101				$CrC_8H_{5.67}O_{5.33}$
Cr-MIL-101a	15.80 ± 0.24	35.05 ± 0.35	3.25 ± 0.14	$CrC_{9.6}H_{10.61}O_8$
Cr-MIL-101b	18.17 ± 0.37	34.35 ± 0.46	2.90 ± 0.15	$CrC_{8.18}H_{8.23}O_{6.64}$
Cr-MIL-101c	20.96 ± 0.17	34.90 ± 0.30	2.80 ± 0.01	$CrC_{7.21}H_{6.89}O_{5.85}$
Cr-MIL-101d	21.37 ± 0.42	36.40 ± 0.40	2.80 ± 0.03	$CrC_{7.37}H_{6.76}O_{5.80}$
**Samples**	**Cr (wt. %)**	**C (wt. %)**	**H (wt. %)**	**MOF content**
Cr-MIL-101@Da-1	2.68 ± 0.00	2.20 ± 0.01	0.20 ± 0.02	16.94 %
Cr-MIL-101@Da-2	3.07 ± 0.00	4.30 ± 0.07	0.30 ± 0.00	16.90 %
Cr-MIL-101@Da-3	4.62 ± 0.00	12.70 ± 0.10	0.80 ± 0.04	22.04 %
Cr-MIL-101@Da-4	6.18 ± 0.00	12.00 ± 0.01	0.90 ± 0.01	28.92 %

ND: The proportion of Cr-MOF content in Cr-MOF@Da is calculated on the basis of Cr content measured by Inductively Coupled Plasma Optical Emission Spectrometer (ICP-OES).

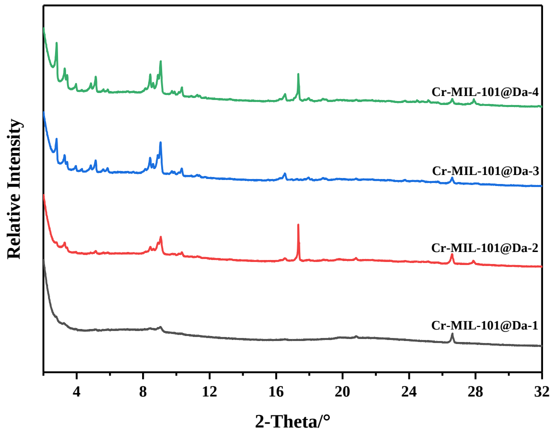

Figure S3. PXRD patterns of Cr-MIL-101@Da-1, Cr-MIL-101@Da-2, Cr-MIL-101 @Da-3 and Cr-MIL-101-@Da-4, respectively.

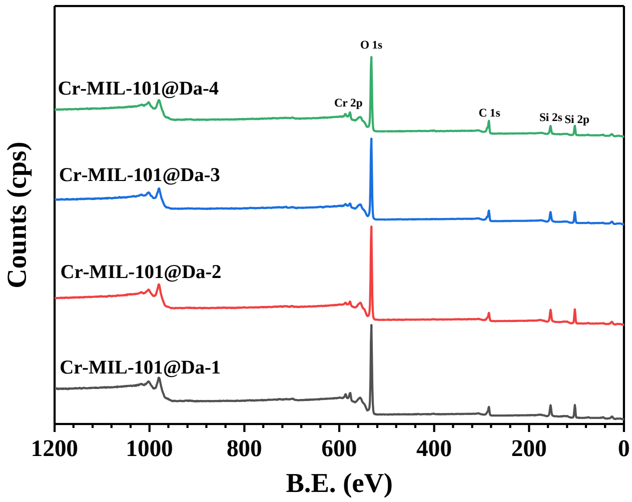

Figure S4. The wide survey scans of Cr-MIL-101@Da-1, Cr-MIL-101@Da-2, Cr-MIL-101 @Da-3 and Cr-MIL-101-@Da-4, respectively.

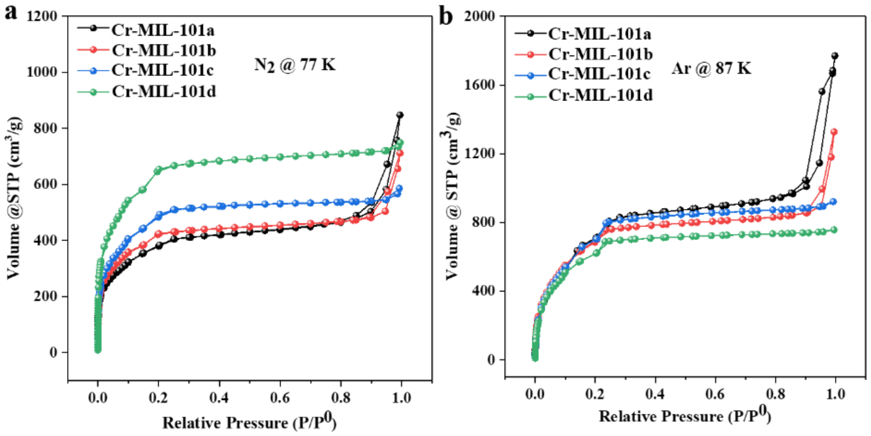

Figure S5. (**a**) N_2 (77 K) and (**b**) Ar (87 K) adsorption-desorption isotherms for Cr-MIL-101 samples demonstrating tunability on porosities of Cr-MIL-101.

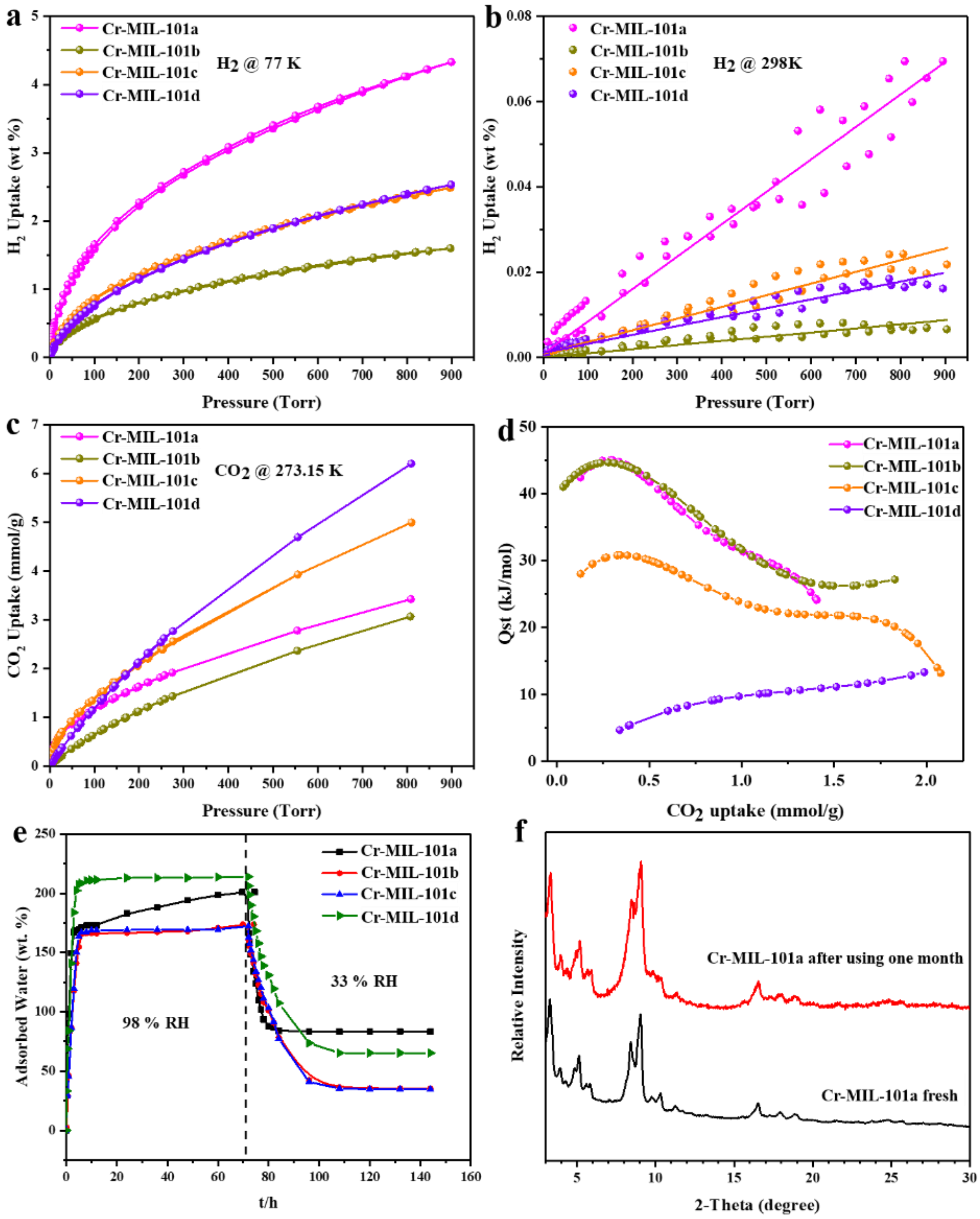

Figure S6. Adsorptive performance of Cr-MOFs. (**a**,**b**) H_2 sorption-desorption isotherms at liquid nitrogen (77 K) and ambient (298 K) temperatures, (**c**) CO_2 sorption-desorption isotherms at 273.15 K, (**d**) CO_2 adsorption enthalpies, (**e**) water adsorption-desorption curves from relative humidity of 98% to 33 % at 298 K and (**f**) comparison of XRD patterns between fresh and used Cr-MIL-101a exposed with 98% RH – 33% RH vapor pairs for over one month.

Table S3. Total H_2 uptake of the obtained Cr-MIL-101@Da hierarchical structures in comparison to the best MOF materials reported so far.

Materials	Temperature (K)	Pressure (bar)	H_2 Uptake (wt. %)	Literature
Cr-MIL-101@Da-3	77	1	5.90 [a]	This work
	298	1	0.09 [b]	
Al-soc-MOF-1	77	1	1.5	[1]
MOF-5	77	1	4.5	[2]
MOF-102	77	35	1	[3]
MOF-505	77	1	2.47	[4]
MOF-508	77	1	0.8	[5]
Mn-BTT	77	1.2	2.2	[6]
CUK-1	77	1	1.60	[7]
CUK-2	77	1	0.66	[7]
PCN-5	77	1	0.63	[8]
PCN-6	77	1	1.9	[9]
PCN-6'	77	1	1.1	[10]
PCN-9	77	1	1.53	[11]
PCN-13	77	1	0.41	[12]
ZIF-8	77	1	1.27	[13]
ZIF-11	77	1	1.35	[13]
IRMOF-3	77	1	1.42	[14]
IRMOF-18	77	1	0.88	[14]
IRMOF-20	77	1	1.32	[14]
HKUST-1	77	1	2.18	[15]

ND: *a* and *b* are calculated in terms of Cr content taking account of 22.04 wt. % of Cr-MIL-101 in composites.

Table S4. Total CO_2 uptake of the obtained Cr-MIL-101@Da materials in comparison to the best MOF materials reported so far.

Materials	Temperature (K)	Pressure (bar)	CO_2 Uptake (mmol/g)	Literature
**Cr-MIL-101@Da-4**	273	1	4.5 [a]	This work
	298	1	3.4 [b]	
**Al-soc-MOF-1**	273	1	1.75	[1]
**MOF-74**	313	0.15	5.28	[16]
**MOF-505**	1	298	2.9	[17]
**Cr-MIL-101-SO_3H**	293	0.4	1.12	[18]
**NbOFFIVE-1-Ni**	298	1	1.3	[19]
**CuBDC**	273	1	1.0	[20]
**Cu-BTG**	273	1	6.39	[21]
**NH_2-MIL-53(Al)**	295	1	1.6	[22]
**MIL-69-Al**	295	1	1.5	[22]
**MIL-96-Al**	295	1	3.6	[22]
**ZIF-94**	295	1	2.3	[22]

ND: *a* and *b* are calculated in terms of Cr content taking account of 28.92 wt. % of Cr-MOF in composites.

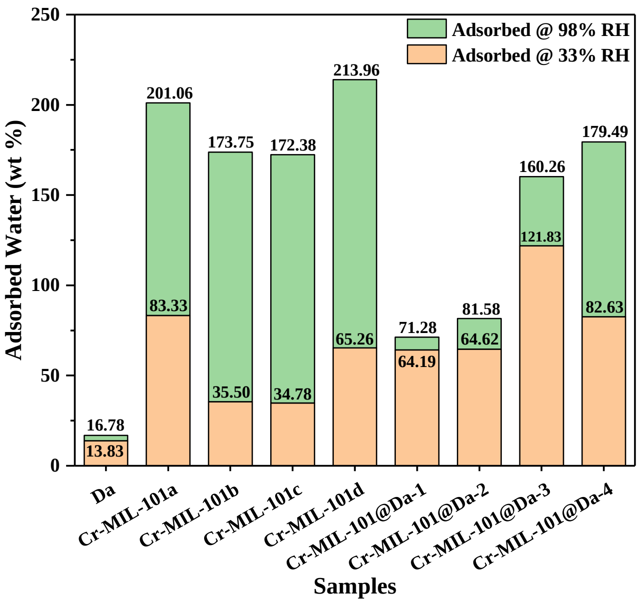

Figure S7. Water adsorption and retaining capacities of various adsorbents from RH 98 % to 33 % at 298 K.

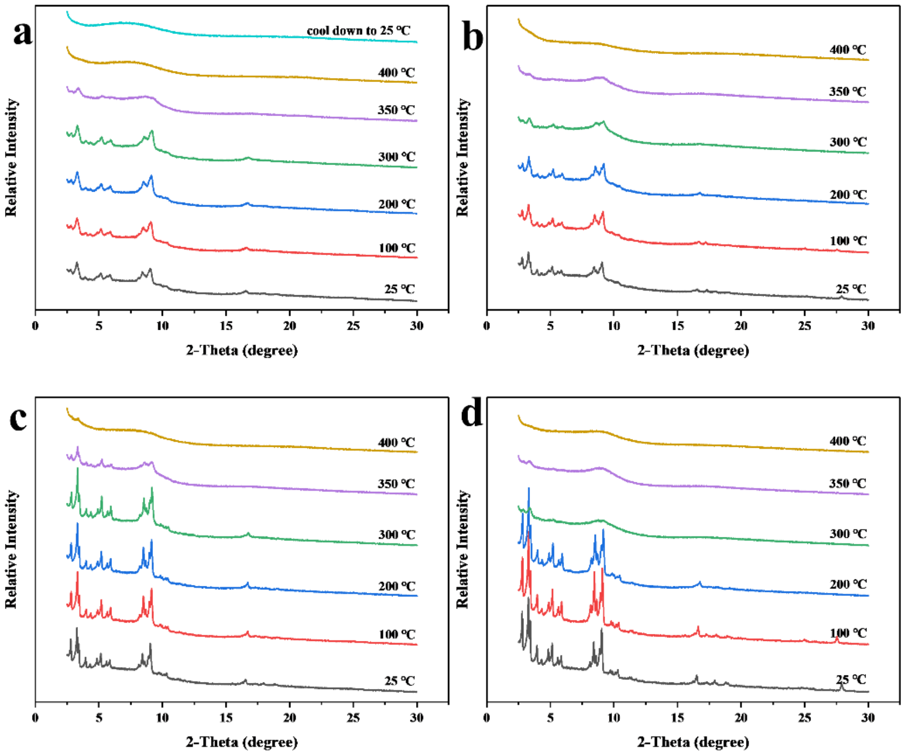

Figure S8. VTPXRD patterns of (**a**) the decomposition of Cr-MIL-101a occurs between 300 and 350 °C, (**b**) the decomposition of Cr-MIL-101b occurs between 200 and 300 °C, (**c**) the decomposition of Cr-MIL-101c occurs between 300 and 350 °C and (**d**) the decomposition of Cr-MIL-101d occurs between 200 and 300 °C.

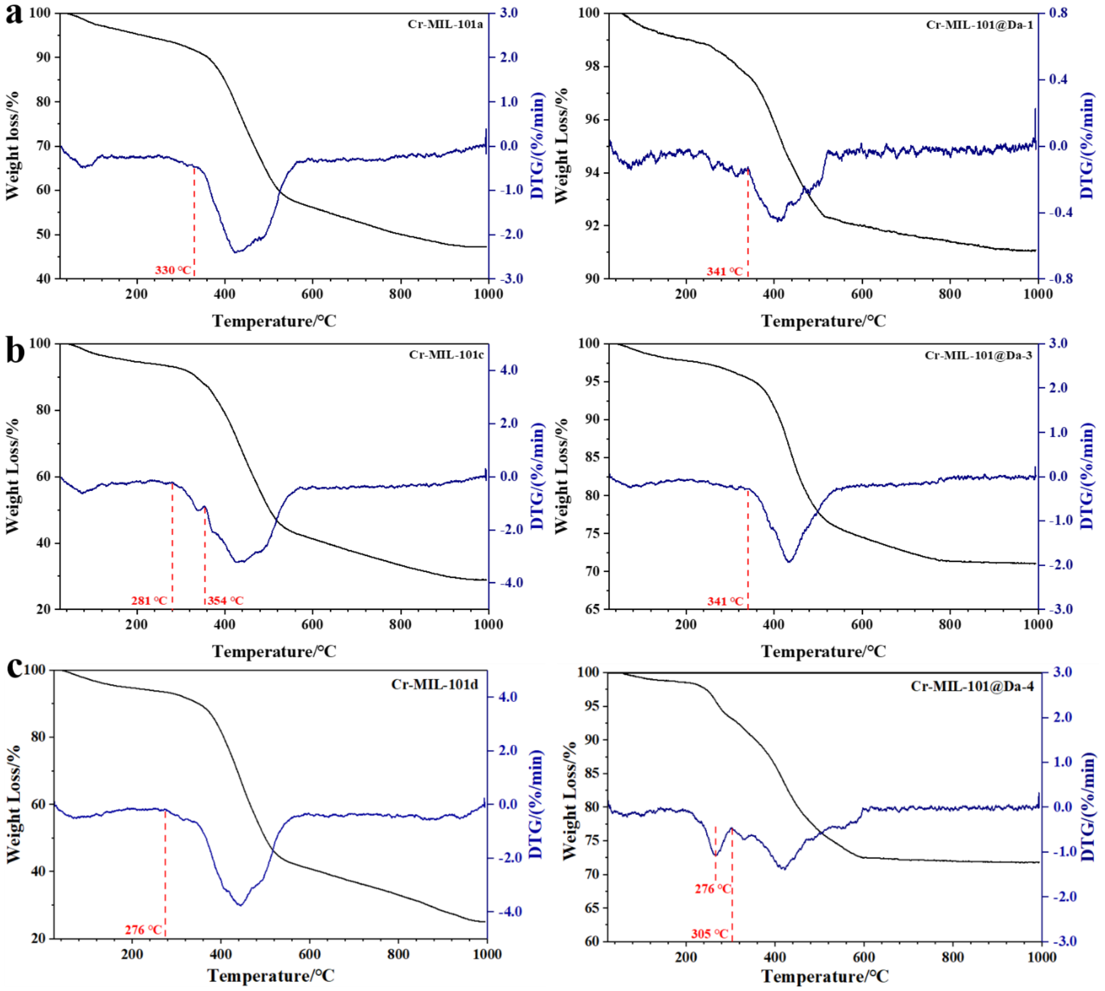

Figure S9. TG-DTG curves of (**a**) left: Cr-MIL-101a and right: Cr-MIL-101@Da-1, (**b**) left: Cr-MIL-101c and right: Cr-MIL-101@Da-3, (**c**) left: Cr-MIL-101d and right: Cr-MIL-101@Da-4.

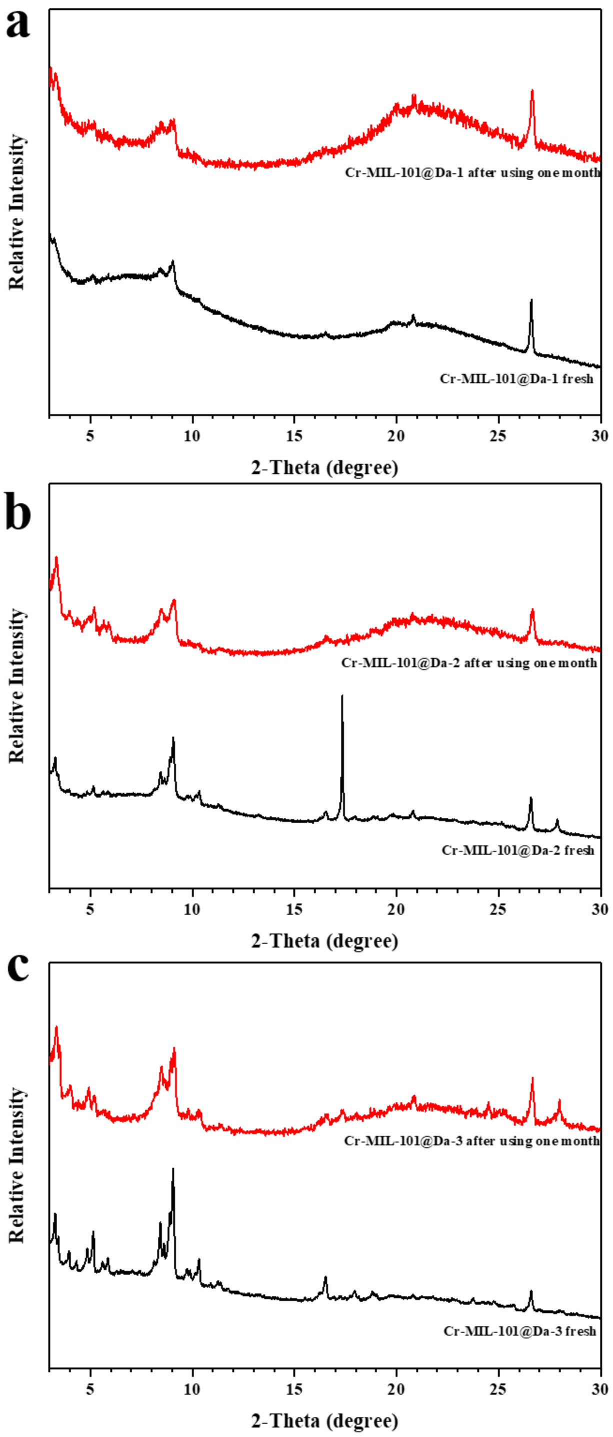

Figure S10. Comparison of XRD patterns between fresh Cr-MIL-101@Da (black) and Cr-MIL-101@Da exposed to 98% RH – 33% RH vapor pairs for one month (red) for (**a**) Cr-MIL-101@Da-1, (**b**) Cr-MIL-101@Da-2 and (**c**) Cr-MIL-101@Da-3, respectively.

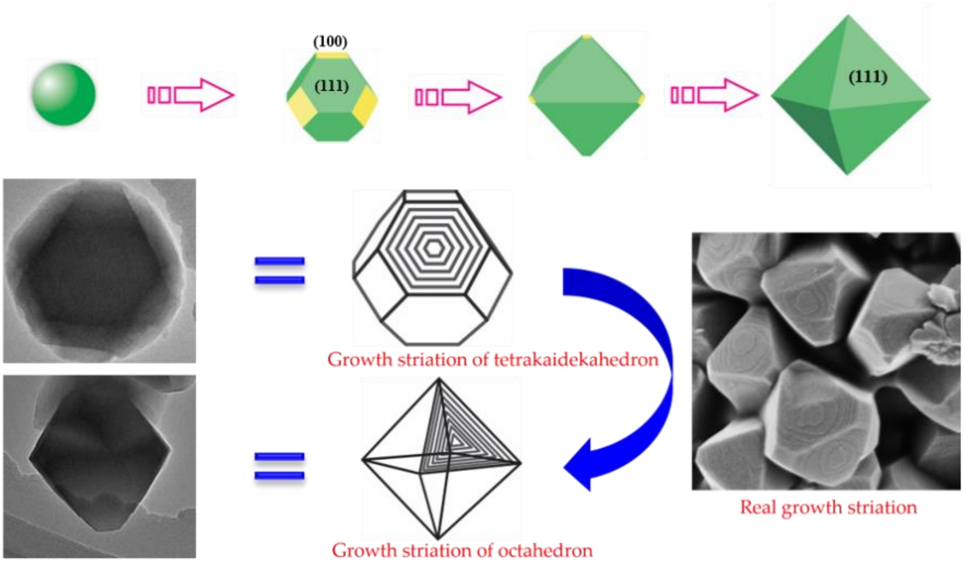

Figure S11. Transformation mechanism for Cr-MIL-101.

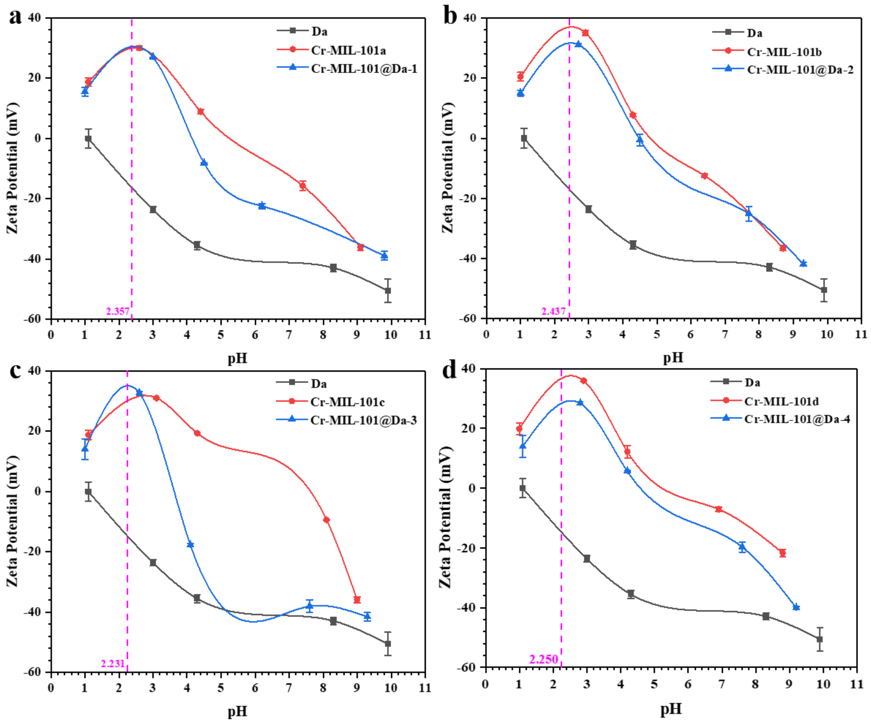

Figure S12. Zeta potentials of Da, Cr-MIL-101 and Cr-MIL-101@Da at various solution pH.

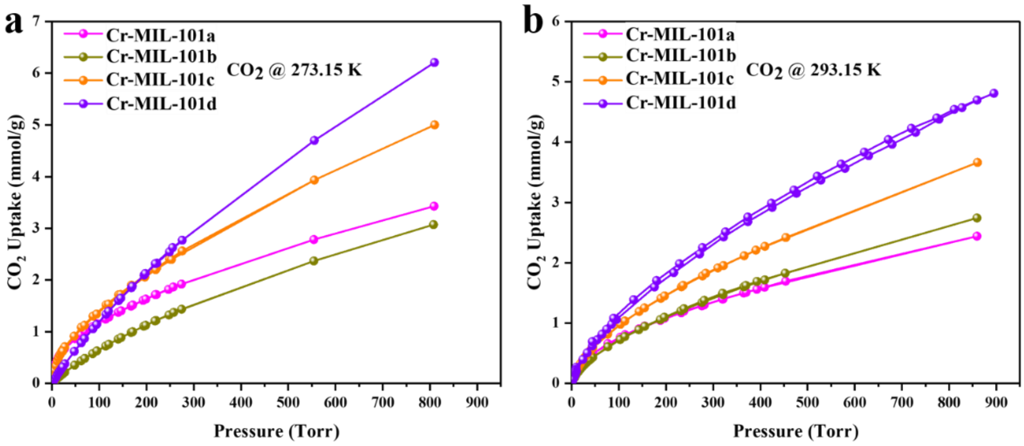

Figure S13. The gravimetric adsorption capacities of Cr-MIL-101 for CO_2 at (a) 273.15 K and (b) 293.15 K.

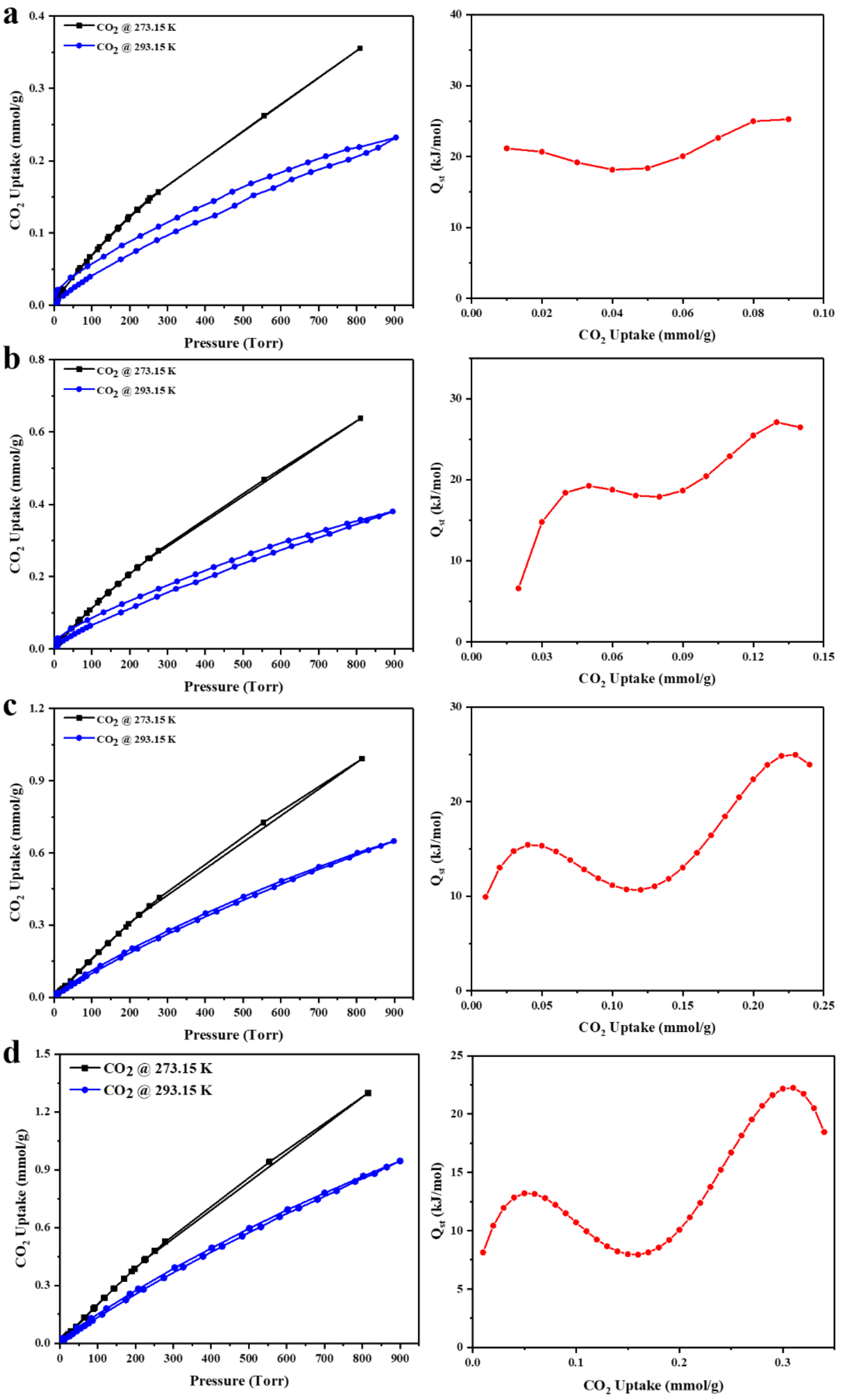

Figure S14. CO_2 adsorption-desorption isotherms (left) and isometric adsorption heat (Q_{st}) of CO_2 calculated from the corresponding adsorption isotherms (right) for (**a**) Cr-MIL-101@Da-1, (**b**) Cr-MIL-101@Da-2, (**c**) Cr-MIL-101@Da-3 and (**d**) Cr-MIL-101@Da-4.

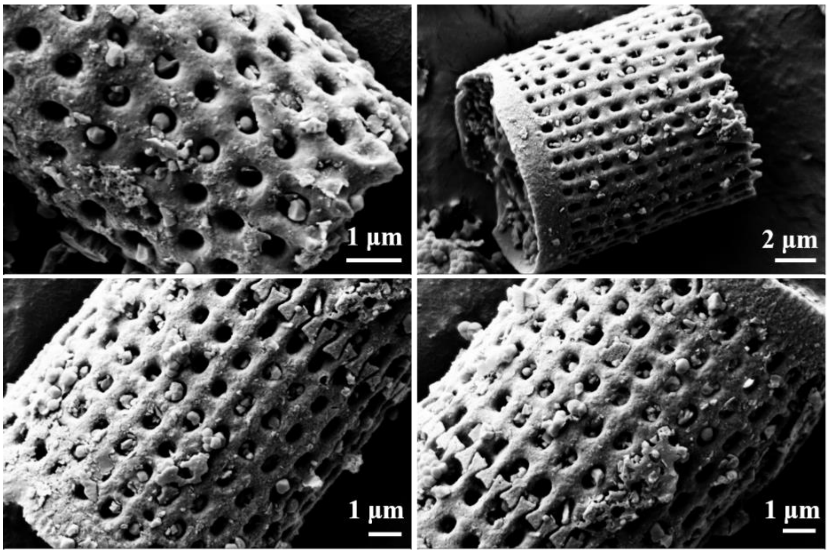

Figure S15. SEM images of Cr-MIL-101@Da-3 after using for two months.

References

1. Alezi, D.; Belmabkhout, Y.; Suyetin, M.; Bhatt, P. M.; Weselinski, L. J.; Solovyeva, V.; Adil, K.; Spanopoulos, I.; Trikalitis, P. N.; Emwas, A. H.; Eddaoudi, M., MOF Crystal Chemistry Paving the Way to Gas Storage Needs: Aluminum-Based soc-MOF for CH4, O2, and CO2 Storage. *J Am Chem Soc* **2015,** 137, (41), 13308-18.
2. Rosi, N. L.; Eckert, J.; Eddaoudi, M.; Vodak, D. T.; Kim, J.; O'Keeffe, M.; Yaghi, O. M., Hydrogen Storage in Microporous Metal-Organic Frameworks. *Science* **2003,** 300, (5622), 1127-1129.
3. Surblé, S.; Millange, F.; Serre, C.; Düren, T.; Latroche, M.; Bourrelly, S.; Llewellyn, P. L.; Férey, G., Synthesis of MIL-102, a chromium carboxylate metal− organic framework, with gas sorption analysis. *Journal of the American Chemical Society* **2006,** 128, (46), 14889-14896.
4. Chen, B.; Ockwig, N. W.; Millward, A. R.; Contreras, D. S.; Yaghi, O. M., High H2 adsorption in a microporous metal–organic framework with open metal sites. *Angewandte Chemie International Edition* **2005,** 44, (30), 4745-4749.
5. Chen, B.; Liang, C.; Yang, J.; Contreras, D. S.; Clancy, Y. L.; Lobkovsky, E. B.; Yaghi, O. M.; Dai, S., A Microporous Metal–Organic Framework for Gas-Chromatographic Separation of Alkanes. *Angewandte Chemie International Edition* **2006,** 45, (9), 1390-1393.
6. Dinca, M.; Dailly, A.; Liu, Y.; Brown, C. M.; Neumann, D. A.; Long, J. R., Hydrogen storage in a microporous metal− organic framework with exposed Mn2+ coordination sites. *Journal of the American Chemical Society* **2006,** 128, (51), 16876-16883.
7. Humphrey, S. M.; Chang, J. S.; Jhung, S. H.; Yoon, J. W.; Wood, P. T., Porous Cobalt (II)–Organic Frameworks with Corrugated Walls: Structurally Robust Gas-Sorption Materials. *Angewandte Chemie* **2007,** 119, (1-2), 276-279.
8. Ma, S.; Wang, X.-S.; Manis, E. S.; Collier, C. D.; Zhou, H.-C., Metal− Organic Framework Based on a Trinickel Secondary Building Unit Exhibiting Gas-Sorption Hysteresis. *Inorganic chemistry* **2007,** 46, (9), 3432-3434.
9. Sun, D.; Ma, S.; Ke, Y.; Collins, D. J.; Zhou, H.-C., An Interweaving MOF with High Hydrogen Uptake. *Journal of the American Chemical Society* **2006,** 128, (12), 3896-3897.
10. Ma, S.; Sun, D.; Ambrogio, M.; Fillinger, J. A.; Parkin, S.; Zhou, H.-C., Framework-catenation isomerism in metal− organic frameworks and its impact on hydrogen uptake. *Journal of the American Chemical Society* **2007,** 129, (7), 1858-1859.
11. Ma, S.; Zhou, H.-C., A Metal−Organic Framework with Entatic Metal Centers Exhibiting High Gas Adsorption Affinity. *Journal of the American Chemical Society* **2006,** 128, (36), 11734-11735.
12. Ma, S.; Wang, X.-S.; Collier, C. D.; Manis, E. S.; Zhou, H.-C., Ultramicroporous Metal−Organic Framework Based on 9,10-Anthracenedicarboxylate for Selective Gas Adsorption. *Inorganic Chemistry* **2007,** 46, (21), 8499-8501.

13. Park, K. S.; Ni, Z.; Côté, A. P.; Choi, J. Y.; Huang, R.; Uribe-Romo, F. J.; Chae, H. K.; O'Keeffe, M.; Yaghi, O. M., Exceptional chemical and thermal stability of zeolitic imidazolate frameworks. *Proceedings of the National Academy of Sciences* **2006,** 103, (27), 10186-10191.
14. Rowsell, J. L.; Yaghi, O. M., Effects of functionalization, catenation, and variation of the metal oxide and organic linking units on the low-pressure hydrogen adsorption properties of metal− organic frameworks. *Journal of the American Chemical Society* **2006,** 128, (4), 1304-1315.
15. Murray, L. J.; Dincă, M.; Long, J. R., Hydrogen storage in metal–organic frameworks. *Chemical Society Reviews* **2009,** 38, (5), 1294-1314.
16. Mason, J. A.; Sumida, K.; Herm, Z. R.; Krishna, R.; Long, J. R., Evaluating metal–organic frameworks for post-combustion carbon dioxide capture via temperature swing adsorption. *Energy & Environmental Science* **2011,** 4, (8), 3030-3040.
17. Chen, Y.; Lv, D.; Wu, J.; Xiao, J.; Xi, H.; Xia, Q.; Li, Z., A new MOF-505@GO composite with high selectivity for CO2/CH4 and CO2/N2 separation. *Chemical Engineering Journal* **2017,** 308, 1065-1072.
18. Li, H.; Wang, K.; Feng, D.; Chen, Y.-P.; Verdegaal, W.; Zhou, H.-C., Incorporation of alkylamine into metal-organic frameworks through a brønsted acid-base reaction for CO2 capture. *ChemSusChem* **2016,** 9, (19).
19. Bhatt, P. M.; Belmabkhout, Y.; Cadiau, A.; Adil, K.; Shekhah, O.; Shkurenko, A.; Barbour, L. J.; Eddaoudi, M., A Fine-Tuned Fluorinated MOF Addresses the Needs for Trace CO2 Removal and Air Capture Using Physisorption. *Journal of the American Chemical Society* **2016,** 138, (29), 9301-9307.
20. Rodenas, T.; Luz, I.; Prieto, G.; Seoane, B.; Miro, H.; Corma, A.; Kapteijn, F.; Llabrés i Xamena, F. X.; Gascon, J., Metal–organic framework nanosheets in polymer composite materials for gas separation. *Nature Materials* **2015,** 14, (1), 48-55.
21. Liu, S.; Sun, L.; Xu, F.; Zhang, J.; Jiao, C.; Li, F.; Li, Z.; Wang, S.; Wang, Z.; Jiang, X.; Zhou, H.; Yang, L.; Schick, C., Nanosized Cu-MOFs induced by graphene oxide and enhanced gas storage capacity. *Energy & Environmental Science* **2013,** 6, (3), 818.
22. Sabetghadam, A.; Liu, X.; Benzaqui, M.; Gkaniatsou, E.; Orsi, A.; Lozinska, M. M.; Sicard, C.; Johnson, T.; Steunou, N.; Wright, P. A., Influence of Filler Pore Structure and Polymer on the Performance of MOF-Based Mixed-Matrix Membranes for CO2 Capture. *Chemistry–A European Journal* **2018,** 24, (31), 7949-7956.

© 2020 by the authors. Submitted for possible open access publication under the terms and conditions of the Creative Commons Attribution (CC BY) license (http://creativecommons.org/licenses/by/4.0/).
